# A Decoy‐Receptor‐Armed Biomimetic Nanotherapeutic With Inherent Tropism for Conserved Pathogenic Macrophages for Treating Osteoarthritis and Intervertebral Disc Degeneration

**DOI:** 10.1002/advs.77171

**Published:** 2026-08-20

**Authors:** Fudong Li, Bin Zhang, Zhiqiu Zhang, Bing Zheng, Linhui Han, Chen Yan, Jialin Li, Kaiqiang Sun, Tianyi Zhao, Jiangang Shi

**Affiliations:** ^1^ Department of Orthopedic Surgery Spine Center Shanghai Changzheng Hospital Naval Medical University Shanghai China; ^2^ Department of Orthopedics Naval Medical Center of PLA Navy Medical University Shanghai China

**Keywords:** intervertebral disc degeneration, macrophage, macrophage‐biomimetic nanosystem, NF‐κB pathway, NLRP3, osteoarthritis, pyroptosis

## Abstract

Osteoarthritis (OA) and intervertebral disc degeneration (IVDD) are debilitating musculoskeletal disorders driven by shared inflammatory pathologies, yet lack effective disease‐modifying therapies. Pro‐inflammatory macrophages are central to their pathogenesis, but their selective therapeutic modulation remains a major challenge. Here, we first used single‐cell transcriptomics to identify a conserved, pathogenic interleukin‐1β (IL‐1β)^+^ macrophage signature fueled by a shared nuclear factor‐κB (NF‐κB)‐inflammation axis in human OA and IVDD. We engineered a macrophage‐biomimetic nanosystem (GM1‐dMOF@PTL), a “doppelgänger” nanosystem designed for targeted cellular reprogramming. The platform consists of a Parthenolide (PTL)‐loaded, lysosome‐escaping metal–organic framework (MOF) core camouflaged with inflammatory macrophage membranes, with the IL‐1β decoy receptor, interleukin‐1 receptor type 2 (IL‐1R2), overexpressed. This design enables a multi‐pronged immunomodulatory strategy: the decoy receptor could neutralize extracellular IL‐1β, while the macrophage‐biomimetic surface facilitates homotypic targeting and preferential uptake by inflammatory macrophages. Upon internalization, the nanosystem potently suppresses the NF‐κB/ NLR family pyrin domain containing 3 (N inflammasome axis, effectively disarming the cell's pyroptotic program. Crucially, local administration in mouse models preserved disc and cartilage integrity, resolved inflammation, and provided sustained alleviation of pain hypersensitivity. This study presents a unified, disease‐modifying nanotherapeutic strategy that targets a shared cellular driver by synergistically neutralizing inflammatory signals and reprogramming pathogenic immune cells to attenuate both spine and joint degeneration.

## Graphical abstract

A biomimetic doppelgänger nanosystem neutralizes extracellular inflammatory cytokines and silences intracellular pyroptosis, reprogramming pathogenic macrophages to attenuate both joint and spine degeneration.

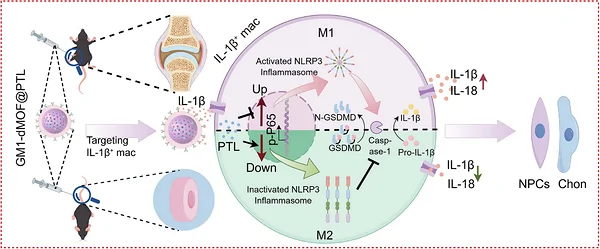

## 1 Introduction

Intervertebral disc degeneration (IVDD) and osteoarthritis (OA) represent two of the most prevalent and debilitating musculoskeletal disorders, collectively inflicting a staggering global burden of chronic pain, functional impairment, and socioeconomic cost [1, 2]. Despite their prevalence, there are currently no disease‐modifying therapies capable of remarkably attenuating or delaying the progression of either condition. Numerous drug candidates have shown preclinical promise, but have consistently failed in clinical trials, largely due to the formidable challenge of achieving sustained, therapeutic concentrations at the site of pathology [3, 4, 5]. The avascular nature of both articular cartilage and the intervertebral discs (IVDs) severely limits drug access from systemic circulation [6, 7]. While local administration via intra‐articular or intra‐discal injection can increase bioavailability, drugs are rapidly cleared, and the dense extracellular matrix (ECM) of both tissues presents a substantial barrier to penetration, preventing therapeutics from reaching their target cells [8, 9]. An ideal therapeutic platform must therefore overcome these shared delivery barriers and engage the core cellular drivers of pathology to elicit a durable biological response.

At their core, IVDs and articular cartilage are architecturally analogous, each comprising specialized cells, nucleus pulposus cells (NPCs) and chondrocytes, respectively, embedded within a dense, proteoglycan‐ and type II collagen‐rich matrix that provides critical biomechanical resilience [10, 11]. This structural parallel gives way to a striking convergence in their pathogenic pathways. Importantly, while the precise etiologies of OA and IVDD remain multifactorial, a growing body of evidence points to chronic, low‐grade inflammation as a central driver of disease progression in both pathologies [12, 13]. In particular, the infiltration and activation of immune cells, most notably macrophages, create a catabolic microenvironment that perpetuates tissue destruction [14, 15]. Activated pro‐inflammatory macrophages secrete a cocktail of destructive enzymes and cytokines, such as matrix metalloproteinases (MMPs), tumor necrosis factor‐alpha (TNF‐α), and interleukin‐1β (IL‐1β), which directly degrade the ECM and induce apoptosis in resident chondrocytes and nucleus pulposus cells [14, 15]. Furthermore, our previous studies have implicated pyroptosis, a highly inflammatory form of programmed cell death mediated by the NLRP3 inflammasome, as a critical mechanism that amplifies the inflammatory response and accelerates tissue degeneration [11, 16, 17]. This suggests that targeting the specific cellular drivers and molecular pathways of this shared inflammatory axis could offer a more fundamental, disease‐modifying therapeutic approach.

The advent of high‐resolution technologies like single‐cell RNA sequencing (scRNA‐seq) has revolutionized our ability to deconstruct the complex cellular ecosystems of diseased tissues [18]. By mapping the transcriptomic landscape at an individual cell level, it is now possible to identify specific pathogenic cell subpopulations and the intercellular signaling networks that orchestrate disease progression. This granular understanding moves beyond targeting broad inflammatory processes and enables the rational design of therapies aimed at the precise cellular culprits. In our present study, scRNA‐seq demonstrated that a conserved, pathogenic pro‐inflammatory macrophage subpopulation orchestrating the NF‐κB‐pyroptosis axis acts as a common pathogenic hub in both diseases. This discovery suggests that targeting this specific cell population and its destructive pyroptotic pathway could represent a unifying and highly effective therapeutic strategy. However, conventional anti‐inflammatory drugs lack the required specificity, often leading to systemic immunosuppression and serious gastrointestinal adverse effects [19, 20]. Their action is akin to indiscriminate systemic blockade, which fails to precisely neutralize the core instigators of the disease while causing significant off‐target toxicity.

**SCHEME 1:**
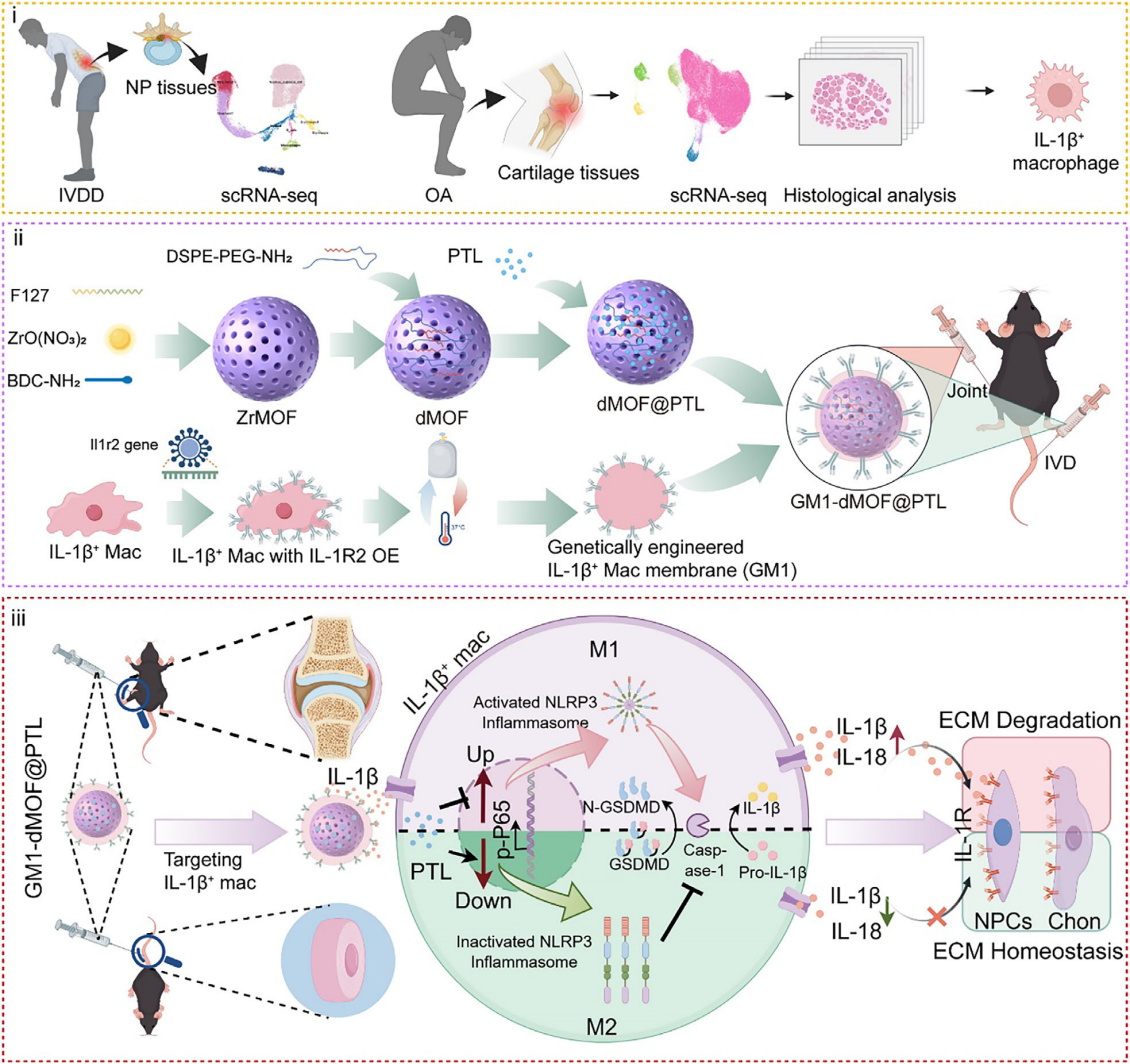
Schematic illustration of the rationale, preparation, and therapeutic mechanism of a “Doppelgänger” nanosystem (GM1‐dMOF@PTL) for treating IVDD and OA. (i) ScRNA‐seq on tissues from IVDD and OA patients reveals a shared pathogenic inflammatory macrophage subpopulation (IL‐1β^+^ macrophage), providing a common therapeutic target for both diseases. (ii) The nanosystem is constructed by first loading a Zr‐based metal–organic framework (ZrMOF) with the NF‐κB inhibitor Parthenolide (PTL). The resulting dMOF@PTL core is then camouflaged with a genetically engineered inflammatory macrophage membrane that overexpresses the decoy receptor IL1R2 (GM1), yielding the final GM1‐dMOF@PTL nanosystem. (iii) In the absence of intervention, pathogenic macrophages undergo NF‐κB/NLRP3‐driven pyroptosis, releasing massive amounts of IL‐1β. This cytokine establishes a paracrine crosstalk with NPCs and chondrocytes, inducing the secretion of matrix‐degrading enzymes and driving ECM destruction. Upon local administration into the joint or intervertebral disc, the GM1 membrane facilitates homotypic targeting to the pathogenic inflammatory macrophages. The nanosystem then enacts a synergistic, two‐pronged attack: the external IL1R2 decoy receptors neutralize extracellular IL‐1β, while the internalized PTL inhibits the intracellular NF‐κB signaling pathway. This dual immunomodulation effectively inactivates the NLRP3 inflammasome, suppresses GSDMD‐mediated pyroptosis, and reduces the secretion of inflammatory cytokines IL1β and IL18. Consequently, the inflammatory macrophages are reprogrammed into a pro‐resolving phenotype, creating a favorable microenvironment that protects nucleus pulposus cells (NPCs) and chondrocytes (Chon) from inflammatory damage, ultimately attenuating the progression of both IVDD and OA.

To address this challenge, we have designed a targeted inflammatory macrophage reprogramming strategy using a multifunctional “doppelgänger” nanosystem, termed GM1‐dMOF@PTL (Scheme 1). We conceptualize this nanosystem not as a simple drug carrier, but as a highly specialized biomimetic platform engineered for precision‐guided cellular modulation. This strategy involves a three‐pronged approach. First, for targeted delivery, the unit is cloaked with genetically engineered pro‐inflammatory macrophage membranes (GM1). This biomimetic surface not only provides biocompatibility and extended circulation but, more importantly, enables homologous targeting to seek out and bind to its pathogenic counterparts in the diseased tissue. Second, to intercept extracellular inflammatory signaling, the engineered membrane is designed to overexpress the IL‐1β decoy receptor (IL1R2), allowing it to act as a molecular sponge that actively neutralizes the key inflammatory signal, IL‐1β, in the microenvironment, thus interrupting the pathogenic amplification cascade. Finally, upon successful entry into the target macrophage, the nanosystem executes its core mission: it releases a “psychological warfare expert,” the payload Parthenolide (PTL), a potent NF‐κB inhibitor and pyroptosis suppressor. PTL directly dismantles the pyroptosis machinery by blocking the NF‐κB/NLRP3 inflammasome axis, effectively disarming the cell's self‐destructive program. Crucially, this intervention not only prevents pyroptosis but also reprograms the macrophage, converting it from a destructive pro‐inflammatory phenotype to a reparative anti‐inflammatory phenotype.

In this study, we demonstrate that the GM1‐dMOF@PTL nanosystem effectively targets pro‐inflammatory macrophages in both IVDD and OA models. It successfully neutralizes extracellular IL‐1β, inhibits pyroptosis, and repolarizes macrophages toward a regenerative state. Consequently, intra‐articular or intradiscal injection of the nanosystem significantly alleviated pain, preserved the cartilage and disc matrix, and restored tissue structural integrity in animal models of OA and IVDD. This work presents a novel therapeutic paradigm that shifts from broad‐spectrum inflammation suppression to targeted cellular reprogramming. By doing so, it offers a precision‐guided strategy to not only pacify the inflammatory insurgency but also convert the primary instigators into agents of repair, holding immense promise as a disease‐modifying treatment for degenerative joint and spine diseases.

## 2 Methods and Materials

### 2.1 Data Acquisition and Processing

Single‐cell RNA sequencing (scRNA‐seq) data for OA and IVDD were sourced from the Gene Expression Omnibus (GEO) repository (http://www.ncbi.nlm.nih.gov/geo). Notably, datasets for OA (GSE220243) and IVDD (GSE165722) were procured [22, 23]. Initial processing entailed rigorous quality control to exclude substandard cells and genes (with counts exceeding 6000 or below 200) and those with a high proportion of mitochondrial/ribosomal genes (>10%). This was followed by normalization via “NormalizeData” and “ScaleData” functions to account for variations in sequencing depth and cell size. Data transformation was implemented to ensure variance stability. For dimensionality reduction, principal component analysis (PCA) was conducted to pinpoint variable genes. The “RunHarmony” function of the Harmony package was utilized to remove batch effects, amalgamating scRNA‐seq data from diverse samples. Subsequent to clustering, the Uniform Manifold Approximation and Projection (UMAP) algorithm was applied for visualization, with cell classification based on the expression of canonical surface markers.

### 2.2 Approximation and Projection Visualization

Uniform Manifold Approximation and Projection, a nonlinear dimensionality reduction technique, was used in single‐cell RNA sequencing (scRNA‐seq) to visualize the high‐dimensional gene expression data in a lower‐dimensional space. The primary goal of UMAP was to preserve the global and local structure of the data while reducing its dimensions, making it easier to identify and interpret patterns, such as cell populations or states. The process began with preprocessing steps, including normalizing the gene expression data and identifying the most variable genes. Then, PCA was applied to reduce the noise and the number of dimensions before UMAP was used. UMAP transforms this high‐dimensional data into a two‐dimensional plot, where each point represents a single cell. Cells with similar expression profiles clustered together, making it possible to visually identify distinct cell populations.

### 2.3 Cluster Annotation

Clustering algorithms were employed to group cells into clusters based on their gene expression profiles. These algorithms worked by partitioning the data into clusters, where cells within the same cluster have more similar expression profiles compared to cells in different clusters. Once clusters were identified, they were annotated by comparing the expression of known marker genes for specific cell types. This step involved analyzing the expression of these marker genes within each cluster to determine which cell type or state it represents.

### 2.4 Pseudotime Analysis

In this study, pseudotime analysis was performed using Monocle2 to infer the temporal progression of cellular states from single‐cell RNA sequencing (scRNA‐seq) data. Initially, scRNA‐seq data was pre‐processed using the Cell Ranger pipeline, which included filtering out low‐quality cells and genes based on specific thresholds. Following quality control, gene expression data was normalized and log‐transformed. Monocle2 was then applied to construct single‐cell trajectories. To achieve dimensionality reduction, Monocle2 uses discriminative dimensionality reduction, which captures the variation in gene expression profiles across cells. Cells were then ordered in pseudotime based on the trajectory, enabling the identification of key transitions and branch points along the differentiation or activation pathways.

### 2.5 Differential Gene Expression Analysis

Differential gene expression analysis was used to identify genes that were significantly upregulated or downregulated between different clusters or conditions. In the context of scRNA‐seq, this involved comparing the gene expression profiles of cells in one cluster against those in another. DESeq2 was used to perform this analysis. The results were visualized in volcano plots, where each point represents a gene. Genes that were significantly different in their expression between the compared groups were highlighted, providing insights into the molecular mechanisms that may differentiate the cell types or conditions.

### 2.6 Transcription Factor Analysis

The Single‐Cell Regulatory Network Inference and Clustering (SCENIC) approach constitutes an analytical technique leveraging co‐expression and motif analysis to delineate gene regulatory networks and to ascertain cellular states from single‐cell RNA sequencing information. Employing the SCENIC tool, we conducted an examination of cell‐type‐specific transcription factors within individual‐cell data arrays. Following the assembly of expression matrices and corresponding annotation files, these data were integrated into the SCENIC application to establish co‐expression networks, transcription factor‐target gene connectivity maps, and to quantify the regulatory activity of transcription factor regulons.

### 2.7 Cell Communication Analysis

Analysis of ligand–receptor interactions serves to delineate cellular communication by pinpointing potential cell‐to‐cell interactions based on the presence of ligands and their matching receptors. Within single‐cell RNA sequencing datasets, this process entails the identification of cells that produce ligands (signal‐emitting molecules) and those that express the associated receptors. Subsequent to this, the interactions amongst these cells are charted to construct a model of communication networks. The CellChat R package (http://www.cellchat.org/) was employed to investigate cellular communication networks derived from single‐cell sequencing information. This integration of single‐cell expression data with cataloged ligands, receptors, and related accessory factors permitted the quantification of intercellular signaling strength. Network analysis and pattern recognition techniques were applied to forecast the principal incoming and outgoing signals of individual cells, as well as the coordinating roles these cells and signals play. The Nitchnet software was used to predict ligand activity by inferring ligand‐target gene relationships in interacting cells, leveraging a combination of cellular expression data, signaling information, and established knowledge of gene regulatory networks.

### 2.8 Heatmap of Gene Expression

Heatmaps are a powerful way to visualize the expression levels of multiple genes across different cell clusters or conditions. In scRNA‐seq analysis, heatmaps display the expression of selected genes (differentially expressed or marker genes) across all cells or clusters. Each row of the heatmap represents a gene, and each column represents a cell or cluster. The intensity of the color in each cell of the heatmap corresponds to the expression level of the gene, with color scales ranging from low to high. Heatmaps include hierarchical clustering of genes and/or cells, which groups together genes or cells with similar expression patterns, making it easier to identify co‐expressed genes and similar cell states.

### 2.9 Molecular Docking

The three‐dimensional (3D) crystal structure of the target protein, P65, was obtained from the Protein Data Bank (PDB ID: 1NFI). The molecular structure of the ligand, (−)‐Parthenolide (PTL), was retrieved from the PubChem database. Prior to docking, the p65 protein structure was pre‐processed using PyMOL (v2.3.0) to remove water molecules and the original co‐crystallized ligand. Concurrently, the 3D structure of PTL underwent molecular mechanics optimization using Chem3D (2020 version) to obtain its most stable, energy‐minimized conformation. For the docking procedure, both the prepared receptor and ligand were converted to the PDBQT file format using AutoDockTools (v1.5.6). Molecular docking was subsequently carried out using AutoDock Vina (v1.2.0) with a semi‐flexible docking protocol, where the protein was held rigid, and the ligand was flexible. The simulation utilized a Lamarckian genetic algorithm with an exhaustiveness parameter set to 8, generating a maximum of nine output conformations for analysis.

### 2.10 Human Samples

This study was conducted in accordance with the Declaration of Helsinki and received ethical approval from the Institutional Review Board of Shanghai Changzheng Hospital, Naval Medical University (No. CZ2023R0253). All participants provided written informed consent prior to inclusion. Human NP tissues were procured intraoperatively from patients undergoing spine surgery for conditions including lumbar disc herniation or tethered cord syndrome. Patients with a history of spinal infection, tumor, or previous spinal surgery at the index level were excluded. Preoperative magnetic resonance imaging (MRI) was used to classify the degree of IVDD according to the Pfirrmann grading system. Patients were stratified into two groups based on degeneration severity: normal, Pfirrmann Grade I or II (n = 5; male = 3, female = 2; mean age 32.1 ± 13.8 years); IVDD, Grade IV or V (n = 5; male = 2, female = 3; mean age 48.7 ± 12.8 years). Human articular cartilage samples and synovial tissues were obtained from patients undergoing knee surgery for conditions including OA (n = 5; male = 1, female = 4; mean age 63.5 ± 9.1 years) or traumatic injury (n = 5; male = 3, female = 2; mean age 31.8 ± 7.3 years) undergoing knee surgery. The radiological severity of OA was evaluated using the Kellgren‐Lawrence (KL) grading system. Furthermore, patients with concurrent severe systemic inflammatory or autoimmune diseases were strictly excluded from both cohorts. Detailed individual clinical demographics, including specific radiological grades and exclusion criteria verification for all recruited subjects, are summarized in supplementary table 2. All tissue samples were collected under aseptic conditions, immediately placed in sterile, pre‐chilled phosphate‐buffered saline (PBS) (Cat. No. G4202, Servicebio, China), and transported to the laboratory for processing.

### 2.11 Isolation and Culture of Bone Marrow‐Derived Macrophages (BMDMs)

All animal experiments and reporting were conducted in strict adherence to the Animal Research: Reporting of In Vivo Experiments (ARRIVE) guidelines. All animal procedures were reviewed and approved by the Institutional Animal Care and Use Committee (IACUC) of Zhejiang Center of Laboratory Animals (ZICLA) (Approval No. ZJCLA‐IACUC‐20050101). Bone marrow cells were isolated from the femurs of 8‐week‐old male C57BL/6 mice provided by GemPharmatech Co., Ltd, Nanjing, China, following protocols adapted from a previous study [21]. Briefly, the bone marrow was flushed from the femurs using Dulbecco's Modified Eagle Medium (DMEM) (Cat. No. 11965092, ThermoFisher Scientific) and passed through a 70‐µm cell strainer to generate a single‐cell suspension. Erythrocytes were depleted by incubating the cell suspension with Red Blood Cell (RBC) Lysing Buffer (Cat. No. C3702, Beyotime, China) according to the manufacturer's instructions. The remaining bone marrow precursor cells were seeded at a density of 5 × 10^6^ cells per dish. Cells were cultured in complete DMEM supplemented with 10% fetal bovine serum (FBS) (Cat. No. C0226, Beyotime, China), 1% penicillin–streptomycin (Cat. No. C0222, Beyotime, China), and 10 ng/mL recombinant murine macrophage colony‐stimulating factor (M‐CSF) (Cat. No. CB34, Novoprotein, China). The cultures were maintained in a humidified incubator at 37°C and 5% CO_2_. On day 3, an additional volume of fresh complete medium with M‐CSF was added. After 7 days of differentiation, the adherent, mature BMDMs were harvested for subsequent experiments.

### 2.12 Isolation and Culture of Primary NPCs and Chondrocytes

We isolated primary NPCs and articular chondrocytes from the tail vertebrae and femoral condyles, respectively, of 8‐week‐old C57BL/6 male mice provided by GemPharmatech Co., Ltd, Nanjing, China. To minimize biological variability and batch effects, all animals were littermates of the same generation and housed within the same cage, with selection performed in a randomized and blinded manner approved by the Animal Care and Use Committee. After aseptic collection, the NP and cartilage tissues were minced and separately digested with 0.25% type II collagenase for 4 h at 37°C to liberate the cells from the extracellular matrix. Subsequently, digestion was terminated, and the mixtures were centrifuged to obtain cell pellets. These cell pellets were then cultured in DMEM/F‐12 supplemented with 10% FBS and 1% penicillin‐streptomycin (P/S) and maintained in a 37°C, 5% CO2 incubator. Crucially, to validate culture purity and health, all cells were confirmed negative for mycoplasma contamination within one week prior to experimentation. When the NPCs and chondrocytes reached 70–80% confluence (Passage 2), they were harvested using trypsin‐EDTA for use in the transwell co‐culture systems to assess ECM homeostasis.

To simulate the in vivo paracrine inflammatory microenvironment and evaluate the protective efficacy of the nanosystem, a non‐contact transwell co‐culture system was established using six‐well transwell inserts with a 0.4 µm pore size polycarbonate membrane (Corning, USA). Primary NPCs or chondrocytes were seeded in the lower chambers at a density of 2 × 10^5^ cells/well and allowed to adhere overnight. Concurrently, macrophages (1 × 10^5^ cells/insert) were seeded into the upper chambers. Following polarization into the M1 pro‐inflammatory phenotype, the upper inserts containing macrophages were transferred to the multi‐well plates containing the NPCs/chondrocytes. The co‐culture systems were then divided into four groups and subjected to respective treatments in the upper or lower chamber: (1) M0 macrophages + vehicle (Control); (2) M1 macrophages + DMSO; (3) M1 macrophages + R‐7050 (a specific TNF receptor antagonist, 5 µM; for the lower chamber); and (4) M1 macrophages + GM1‐dMOF@PTL (for the upper chamber). After 48 h of co‐culture, the upper inserts were removed, and the primary NPCs or chondrocytes in the lower chambers were harvested for RNA extraction and subsequent RT‐qPCR analysis to assess the mRNA levels of *Col2a1* and *Mmp13*.

### 2.13 Lentiviral Vector Construction and Transduction of BMDMs

Recombinant lentiviruses for the overexpression of murine IL1R2, as well as a corresponding blank vector lentivirus, were purchased from Shanghai Genechem Co., Ltd. (Shanghai, China). The transduction of BMDMs was carried out in accordance with the lentivirus infection manual provided by the manufacturer. The overexpression of IL1R2 in the resulting cell population was confirmed to ensure the effectiveness of the transduction.

### 2.14 Plasmid Transfection and In Vitro Rescue Assay

To validate whether the nanosystem alleviates macrophage inflammation and pyroptosis strictly through the NF‐κB signaling pathway, a P65‐overexpression (P65‐OE) macrophage model was established. Macrophages were seeded into 24‐well plates containing glass coverslips at a density of 3 × 10^5^ cells per well and cultured overnight. A pcDNA3.1 plasmid carrying the full‐length murine p65 gene was purchased from GenePharma (Shanghai, China). Transient transfection was performed using Lipofectamine 2000 (Invitrogen, USA) according to the manufacturer's protocol. Briefly, 500 ng of the recombinant p65 plasmid (or empty pcDNA3.1 vector as the negative control) and the Lipofectamine reagent were diluted in Opti‐MEM, mixed, and added to the cells. After 6 h of incubation, the transfection medium was replaced with fresh complete culture medium, and the cells were cultured for an additional 24 h to ensure sufficient P65 protein expression. Following successful transfection, the P65‐OE macrophages were divided into three groups and treated with fresh medium containing equivalent volumes of DMSO (vehicle control), 25 µM of JSH‐23 (a specific NF‐κB inhibitor), or GM1‐dMOF@PTL.

### 2.15 Construction of GM1‐dMOF@PTL

The c‐based metal–organic framework (ZrMOF) was prepared first. Initially, 250 mg of the surfactant Pluronic F127 was dissolved in 10 mL of deionized water by stirring at 60°C for 10 min to promote nucleation and crystallinity. Subsequently, 2 mL of acetic acid was introduced as a modulator. A solution of sodium perchlorate (1 g in 1 mL of water) was then added to the reaction mixture, which was stirred for an additional 10 min. The zirconium precursor, prepared by dispersing 578 mg of zirconium oxynitrate in 2 mL of water, was then added to the system, followed by 5 min of stirring. Finally, 200 mg of the organic linker, 2‐aminoterephthalic acid (BDC‐NH2), was added. The reaction was maintained at 60°C for 12 h under continuous magnetic stirring. The resulting product was harvested by centrifugation (13,000 × g, 5 min) and subjected to a sequential purification process. The precipitate was washed once with deionized water, followed by a wash with N,N‐dimethylformamide (DMF). The material was then heated in ethanol at 60°C for 6 h. This ethanol‐heating step was repeated a second time, with the product being collected by centrifugation after each cycle. The purified ZrMOF was dispersed in 20 mL of ethanol and stored for subsequent experimental use.

The surface of the ZrMOF nanoparticles was functionalized with DSPE‐PEG‐NH_2_ using a thin‐film hydration method. Briefly, a solution of DSPE‐PEG‐NH_2_ in chloroform (1 mg/mL) was added to a round‐bottom flask. A uniform lipid film was formed on the inner wall of the flask by removing the organic solvent via rotary evaporation at 45°C, followed by drying under vacuum for at least 2 h to eliminate any residual solvent. The resulting lipid film was then hydrated with a 1 mg/mL aqueous dispersion of the as‐prepared ZrMOF nanoparticles. The mixture was incubated at 50°C for 1 h under continuous magnetic stirring to facilitate the self‐assembly and insertion of the DSPE‐PEG‐NH_2_ molecules onto the surface of the ZrMOF. Finally, to remove any free, unconjugated DSPE‐PEG‐NH_2_ molecules, the resulting suspension was purified by dialysis against ultrapure water for 24 h using a dialysis membrane (MWCO 20 kDa), with the dialysate being replaced every 4 h. The purified DSPE‐PEG‐NH_2_‐modified ZrMOF (d‐MOF) was collected and stored at 4°C for subsequent experiments.

The isolation of macrophage‐derived plasma membranes was conducted utilizing a Membrane and Cytosolic Protein Extraction Kit (Cat. No. P0033, Beyotime, China) following a modified protocol. Briefly, approximately 5 × 10^7^ IL1R2‐overexpressed and LPS‐induced‐inflammatory BMDMs were harvested, washed once with ice‐cold PBS, and collected by centrifugation (800 × g, 5 min, 4°C). The resulting cell pellet was resuspended in 1 mL of Membrane Extraction Reagent A, which had been freshly supplemented with phenylmethylsulfonyl fluoride (PMSF) to a final concentration of 1 mM. The cell suspension was incubated on ice for 15 min to facilitate cell swelling. To lyse the cells, the suspension was subjected to three repetitive freeze–thaw cycles, alternating between immersion in liquid nitrogen for 5 min and subsequent thawing at room temperature. A low‐speed centrifugation step (700 × g, 10 min, 4°C) was then performed to pellet nuclei and intact cellular debris. The supernatant, containing the membrane fraction, was carefully collected. Subsequently, the plasma membrane fragments were isolated by high‐speed centrifugation at 14,000 × g for 30 min at 4°C. The final membrane pellet was resuspended in sterile PBS. The prepared macrophage membrane vesicles were stored at −80°C until use.

A total of 2 mg PTL was initially mixed with 1 mg dMOF in ethanol to ensure drug saturation of the carrier pores. The mixture was incubated under gentle stirring for 12 h at room temperature to facilitate maximal drug loading. Excess, unbound PTL was then thoroughly removed by centrifugation (13,000 × g, 10 min) and subsequent triple washes with PBS. A macrophage membrane solution containing 1 mg of total membrane protein was mixed with 1 mg of the PTL‐loaded dMOF@PTL nanoparticles, establishing a core‐to‐membrane protein weight ratio of 1:1. This mixture was then sonicated and subsequently processed by extrusion through a 200 nm polycarbonate membrane for 11 passes to form a uniform membrane coating. To remove unincorporated membrane vesicles, the sample was centrifuged at 11,000 × g for 10 min. The final pellet, consisting of membrane‐coated nanoparticles (GM1‐dMOF@PTL), was collected and resuspended in PBS for subsequent characterization and in vitro/in vivo studies.

### 2.16 Characterization of GM1‐dMOF@PTL

The morphology of the GM1‐dMOF@PTL and dMOF@PTL nanoparticles was examined by transmission electron microscopy (TEM; HT7700, Hitachi, Japan). Samples of GM1‐dMOF@PTL and dMOF@PTL were prepared by depositing a diluted aqueous suspension onto a carbon‐coated copper grid and allowing it to air‐dry before imaging. The hydrodynamic diameter and zeta potential of the nanoparticles dispersed in deionized water were determined using a dynamic light scattering (DLS) instrument (Malvern Instruments Ltd., Malvern, UK). To assess the in vitro drug release profile, GM1‐dMOF@PTL and dMOF@PTL were suspended in PBS and sealed within dialysis bags (MWCO 3500 Da). These bags were immersed in 50 mL of distinct release media: (1) standard PBS at pH 7.4 (simulating normal physiological conditions), and (2) an acidic buffer adjusted to pH 6.5 and supplemented with 10 ng/mL of recombinant IL‐1β (simulating the highly acidic and inflammatory microenvironment of the degenerated joint and disc). The systems were incubated at 37°C with gentle shaking. At predetermined time points over a 7‐day continuous period, 1 mL of the release medium was collected for analysis and replaced with an equal volume of the corresponding fresh medium. The concentration of the released PTL was quantified using high‐performance liquid chromatography (HPLC). Additionally, to visually evaluate the structural integrity and resistance to degradation of the ZrMOF core in the pathological microenvironment, aliquots of the nanoparticles incubated in the physiological (pH 7.4) and the simulated acidic inflammatory (pH 6.5 + 10 ng/mL IL‐1β) buffers were collected after the 7‐day incubation and observed via TEM. The encapsulation efficiency and drug loading capacity were determined by centrifuging the nanoparticle suspension to separate the free drug in the supernatant from the encapsulated drug, with the amount of PTL measured by HPLC.

To evaluate homologous targeting, nanoparticles were fluorescently labeled by FITC. Macrophages, NPCs, AFCs, and chondrocytes were seeded in confocal dishes and incubated with nanoparticles for 4 h. Following incubation, cells were washed, fixed with 4% paraformaldehyde, and stained with DAPI. The cellular uptake was visualized qualitatively using a confocal laser scanning microscope (Leica SP8, Leica, USA). To confirm the essential role of surface membrane proteins in mediating the targeted cellular uptake, a protein digestion experiment was performed according to previously established protocols [22]. Prior to co‐incubation with the cells, the GM1‐dMOF@PTL nanoparticles were pretreated with a 0.25% trypsin solution and incubated for 10 min at 37°C to digest the surface‐exposed membrane proteins. Following this digestion period, the trypsinized nanoparticles were collected via centrifugation (11000 × g, 10 min, 4°C) and washed twice with PBS to completely remove any residual trypsin. These pretreated nanoparticles were then introduced to the pro‐inflammatory M1 macrophages for the standard 4‐h uptake assay as described above.

### 2.17 SDS‐PAGE and Western Blot Analysis

To verify the protein composition of the nanoparticles, samples including the source macrophage membrane lysate, uncoated dMOF@PTL, and membrane‐coated GM1‐dMOF@PTL were lysed in RIPA buffer (Cat. No. P0013B, Beyotime, China) supplemented with a 1% protease inhibitor cocktail. The protein concentration of each lysate was quantified using a BCA Protein Assay Kit (Cat. No. P0009, Beyotime, China). Equal amounts of protein (20 µg) from each sample were loaded and separated on a 10% sodium dodecyl sulfate‐polyacrylamide gel electrophoresis (SDS‐PAGE) gel. For total protein profiling, one gel was stained with Coomassie Brilliant Blue (Cat. No. G2039, Servicebio, China). For Western blot analysis, proteins from a parallel gel were transferred onto a polyvinylidene fluoride (PVDF) membrane (Millipore, USA). The membranes were blocked for 1 h at room temperature with 5% non‐fat milk dissolved in Tris‐buffered saline containing 0.1% Tween‐20 (TBST). Subsequently, the membranes were incubated overnight at 4°C with primary antibodies against IL1R2 (1:1000; Cat. No. 60262‐1‐Ig, Proteintech, China), CD68 (1:1000; Cat. No. 28058‐1‐AP, Proteintech, China), Integrin β1 (1:2000; Cat. No. 12594‐1‐AP, Proteintech, China), Na^+^K^+^‐ATPase (1:1000; Cat. No. AF6109, Affinity, China), P65 (1:1000; Cat. No. AF5006, Affinity, China), and GAPDH (1:1000; Cat. No. AF7021, Affinity, China). After three washes with TBST, the membranes were incubated with the appropriate secondary antibodies (1:5000; Cat. No. GB23303, Servicebio, China) for 1 h at room temperature. Protein bands were visualized using an enhanced chemiluminescence (ECL) substrate kit and captured with the ChemiDoc XRS+ imaging system (Bio‐Rad, USA). To ensure data transparency and reproducibility, all full‐length, uncropped western blot images corresponding to the cropped representative immunoblots shown in this manuscript are provided in the Supplementary Information of western blot images.

### 2.18 Scanning Electron Microscopy (SEM)

To observe ultrastructural changes indicative of pyroptosis, mouse BMDMs were cultured in 10 cm dishes. Following the specified experimental treatments, the samples were prepared for SEM analysis. The cells were first fixed with an electron microscope fixative solution (2.5% glutaraldehyde in 0.1 M phosphate buffer, pH 7.4) for at least 4 h at 4°C. Post‐fixation, the coverslips were rinsed three times with PBS, with each rinse lasting 10 min, to remove residual fixative. Subsequently, the samples underwent dehydration through a graded ethanol series, consisting of sequential 15‐min incubations in 30%, 50%, 70%, 80%, and 95% ethanol. This was followed by two 15‐min incubations in 100% ethanol to ensure complete water removal. The dehydrated samples were then subjected to critical point drying. To prepare them for imaging, the dried samples were mounted on aluminum stubs and coated with a thin layer of gold using an ion sputter coater. The surface morphology of the BMDMs was then visualized and captured using a scanning electron microscope (Gemini SEM 300, Carl Zeiss, Germany).

### 2.19 Cytotoxicity and Biosafety Analysis

The cytotoxicity of the formulations was assessed using a Cell Counting Kit‐8 (CCK8) assay. BMDMs were seeded into 96‐well plates at a density of 1 × 10^4^ cells per well and cultured overnight to allow for adherence. The medium was then replaced with fresh medium containing various concentrations of GM1‐dMOF@PTL (0, 1, 10, 20, 50, 100, 500 µg/mL). After a 24‐h incubation period at 37°C, 10 µL of CCK8 solution (Dojindo, Kumamoto, Japan) was added to each well, and the plates were incubated for an additional 2 h. The absorbance at 450 nm was measured using a microplate reader (Bio‐Rad, USA). Cell viability was calculated as a percentage relative to the untreated control group.

To evaluate systemic biocompatibility, healthy C57BL/6 mice (6–8 weeks old) were randomly divided into four groups (n = 5 per group): DMSO, PTL (0.1 mg/kg), dMOF@PTL (0.465 mg/kg), and GM1‐dMOF@PTL (0.625 mg/kg). The mice were administered local injections of 2 µL of the respective formulations into the intervertebral discs. After 8 weeks, the mice were anesthetized, and blood samples were collected via cardiac puncture. A portion of each blood sample was collected in EDTA‐coated tubes for routine blood analysis, while the remainder was collected in serum separator tubes and centrifuged at 3000 × g for 15 min to obtain serum. Major organs (heart, liver, spleen, lungs, and kidneys) were harvested immediately post‐euthanasia. For histopathological analysis, organs were fixed in 4% paraformaldehyde, embedded in paraffin, sectioned, and stained with H&E for microscopic examination. Serum levels of alanine aminotransferase (ALT), aspartate aminotransferase (AST), blood urea nitrogen (UREA), and creatinine (CREA) were quantified using an automated biochemical analyzer (Chemray 800, China). Hematological parameters, including red blood cell (RBC), white blood cell (WBC), lymphocyte, and neutrophil counts, were measured with an automated hematology analyzer (Mindray, China).

### 2.20 Transcriptome Sequencing Analysis

Total RNA was extracted from macrophages stimulated with LPS (1 µg/mL) and ATP (5 mM) with or without treatment using TRIzol reagent. Following purification, RNA integrity was confirmed (RIN > 7.0) using an Agilent 2100 Bioanalyzer. mRNA was isolated using oligo(dT) magnetic beads, fragmented, and reverse‐transcribed into cDNA. Paired‐end (2 × 150 bp) sequencing libraries were prepared and sequenced on an Illumina NovaSeq 6000 platform (LC‐Bio Technology Co., Ltd.). Raw reads were filtered using fastp, and clean reads were aligned to the Mus musculus reference genome (GRCm38) using HISAT2. Gene expression was quantified as FPKM values using StringTie. Differentially expressed genes (DEGs) were identified using the edgeR package in R, with criteria set at a fold change > 2 and a *p*‐value < 0.05. Gene Ontology (GO) and Kyoto Encyclopedia of Genes and Genomes (KEGG) pathway enrichment analyses were performed on the DEGs. Finally, Gene Set Enrichment Analysis (GSEA) was conducted to identify significantly enriched biological pathways and processes, providing further insight into the molecular mechanisms of the treatment.

### 2.21 Establishment of OA Model in Mice

To establish an OA model in mice, we employed the anterior cruciate ligament transection (ACLT) method. Male C57BL/6J mice, aged 8 weeks and weighing 20–25 g, were used in this study (GemPharmatech Co., Ltd, Nanjing, China). The 8‐week age time point was specifically selected in accordance with OARSI guidelines for murine surgical models. At 8 weeks, the zonal architecture of the articular cartilage and the distinct morphological boundaries of the intervertebral disc are structurally established, and the innate immune system is functionally mature. Furthermore, initiating the 8‐week study duration at this age ensures that the end‐point evaluation occurs at 16 weeks of age, aligning precisely with murine skeletal maturity and peak bone mass. This timeline avoids the confounding effects of age‐related spontaneous degeneration while allowing for the clear differentiation between normal physiological maturation (observed in age‐matched Sham controls) and the severe, pathology‐driven structural collapse induced by the surgical procedures. Mice were anaesthetized with 2% isoflurane delivered in oxygen. Aseptic surgical techniques were maintained throughout the procedure. For the OA group, a medial parapatellar incision was made to expose the knee joint, followed by the complete transection of the anterior cruciate ligament using a microsurgical scalpel. The joint was then stabilized, and the incision was closed with absorbable sutures. Mice were randomly assigned into OA groups with various treatments. Mice in treatment groups were treated with 2 µL DMSO, PTL (0.1 mg/kg), dMOF@PTL (0.465 mg/kg), GM1‐dMOF@PTL (0.625 mg/kg), and celecoxib (10 µg/µl) using a 5 µL Hamilton syringe. A second, identical injection was administered one week later. As for the sham group, the suturing was done immediately after the incision. Postoperative pain management was provided with subcutaneous injections of buprenorphine (0.05 mg/kg). Animals were allowed to recover in their cages with free access to food and water. After 8 weeks, the mice were sacrificed, and the knee joint tissues were harvested. The experiments were blindly conducted by one experienced member of this study group.

### 2.22 Establishment of IVDD Model in Mice

Eight‐week‐old male C57BL/6J mice (GemPharmatech Co., Ltd, Nanjing, China) were acclimated for one week and housed under a 12‐h light/dark cycle with ad libitum access to food and water. To induce annulus fibrosus puncture (AFP)‐induced IVDD model, mice were anesthetized with 2% isoflurane delivered in oxygen. Following aseptic preparation of the surgical site with povidone‐iodine and 70% ethanol, the mice were positioned prone. Under fluoroscopic guidance, the caudal intervertebral disc at the Co7‐8 level was percutaneously punctured with a 26‐gauge needle. The needle was inserted through the annulus fibrosus into the nucleus pulposus, rotated 360°, held in place for 30 s to ensure injury, and then withdrawn. Post‐procedural analgesia was provided via a subcutaneous injection of buprenorphine (0.05 mg/kg). Three days following the initial injury, animals were re‐anesthetized, and a 2 µL volume of the designated reagent such as DMSO, PTL (0.1 mg/kg), dMOF@PTL (0.465 mg/kg), GM1‐dMOF@PTL (0.625 mg/kg), and celecoxib (10 µg/µl) was injected into the center of the injured IVDs using a 5 µL Hamilton syringe fitted with a 33‐gauge needle. A second, identical injection was administered one week later. A sham‐operated group underwent anesthesia and needle insertion without annulus fibrosus puncture. After 8 weeks, the mice were sacrificed, and the intervertebral discs were harvested. The experiments were blindly conducted by one experienced member in this study group.

### 2.23 Tissue Section Staining

Following euthanasia, murine spinal motion segments (n = 5 per group) and whole knee joints (n = 5 per group) were harvested and immediately fixed in 10% neutral buffered formalin for 48 h at 4°C. The specimens were subsequently decalcified in 10% ethylenediaminetetraacetic acid (EDTA) solution (pH 7.4) for 4 weeks, with the solution refreshed twice weekly. After complete decalcification, the tissues were embedded in paraffin. Sections of 5 µm thickness were prepared using a rotary microtome. For morphological assessment, sections were stained with Hematoxylin and Eosin (H&E) to evaluate tissue architecture and cellularity. To assess matrix composition, parallel sections were stained with Safranin O‐Fast Green (SOFG) to visualize proteoglycan content. Stained sections were imaged using a digital microscope (Leica DM4 B, Germany). Histopathological degeneration of the IVDs was quantitatively scored based on a standardized grading system [23]. Cartilage degradation in the knee joints was evaluated using the Osteoarthritis Research Society International (OARSI) scoring system [24]. The methodology for grading synovitis was adapted from a recent report [25], employing a four‐point scale (0, 0.5, 1, and 2) to facilitate a comprehensive assessment of various joint locations. A grade of 0 indicated no evidence of synovitis. Grade 0.5 denoted focal synovial thickening, localized to either the synovial plica adjacent to the joint capsule or the intercondylar region (notch of the distal femur or eminence of the proximal tibia). Grade 1 represented extensive thickening involving both the synovial lining (intima) and subintimal stroma within one of these locations, while grade 2 was assigned when such thickening was observed in both regions. For this evaluation, thickening was defined as a visual increase in synovial thickness exceeding 1.5 times the normal state.

### 2.24 Immunofluorescence Staining

Immunofluorescence staining was used to detect specific proteins in tissue sections and cells. In the present study, the immunofluorescence staining was performed to visualize the expression of proteins. The process began with fixing the tissue sections, followed by blocking to prevent nonspecific binding. Primary antibodies, including P65 (1:200; Cat. No. 10745‐1‐AP, Proteintech, China), Arginase 1 (ARG1, 1:200; Cat. No. 16001‐1‐AP, Proteintech, China), Inducible Nitric Oxide Synthase 2 (NOS2, iNOS, 1:200; Cat. No. 22226‐1‐AP, Proteintech, China), NLR Family Pyrin Domain Containing 3 (NLRP3, 1:200; Cat. No. 30109‐1‐AP, Proteintech, China), type II collagen (COL2A1, 1:200; Cat. No. 28459‐1‐AP, Proteintech, China), matrix metalloproteinase 13 (MMP13, 1:200; Cat. No. 18165‐1‐AP, Proteintech, China), Aggrecan (ACAN, 1:200; Cat. No. DF7561, Affinity, China), and Gasdermin D (GSDMD, 1:200; Cat. No. AF4012, Affinity, China), were then applied to the sections, binding to their respective targets. After washing, fluorescently labeled secondary antibodies, which bind to the primary antibodies, were added. The sections were then visualized under an Olympus AX70 microscope.

### 2.25 Immunohistochemical (IHC) Analysis

Paraffin‐embedded sections (5 µm) were deparaffinized in xylene and rehydrated through a graded ethanol series. For antigen retrieval, slides were immersed in 0.01 M sodium citrate buffer (pH 6.0) and heated in a water bath at 95°C for 20 min. Endogenous peroxidase activity was quenched by incubating the sections in 3% hydrogen peroxide for 15 min, followed by blocking with 5% normal goat serum for 1 h at room temperature to prevent nonspecific antibody binding. The sections were then incubated overnight at 4°C with the following primary antibodies: MMP13 (1:100; Cat. No. 18165‐1‐AP, Proteintech, China), ACAN (1:100; Cat. No. DF7561, Affinity, China), NLRP3 (1:100; Cat. No. 30109‐1‐AP, Proteintech, China), GSDMD (1:100; Cat. No. AF4012, Affinity, China), IL1B (1:100; Cat. No. AF5103, Affinity, China), and IL18 (1:100; Cat. No. DF6252, Affinity, China). Following primary antibody incubation, slides were treated with a horseradish peroxidase (HRP)‐conjugated secondary antibody, and the signal was visualized using a 3,3′‐diaminobenzidine (DAB) substrate kit. Nuclei were counterstained with hematoxylin. The percentage of positively stained cells for each target protein was quantified using ImageJ software (National Institutes of Health) by applying a standardized color deconvolution and intensity threshold.

### 2.26 Reverse Transcription Quantitative Polymerase Chain Reaction (RT‐qPCR)

Total RNA was extracted from the cells using a commercial RNA extraction kit‐ TRIzol Reagent (Cat. No. 15596026CN, Invitrogen, USA) according to the manufacturer's protocol. The purity and concentration of the RNA were determined using a NanoDrop spectrophotometer (Thermo Fisher Scientific, USA), with an absorbance ratio of 260/280 nm being used to assess RNA purity. Then the extracted RNA (1 µg) was reverse‐transcribed into complementary DNA (cDNA) using a reverse transcription kit (Cat. No. RR037A, Takara, China) following the manufacturer's instructions. The cDNA was used as a template for quantitative PCR to amplify the target genes. qPCR was performed using a real‐time PCR detection system (Applied Biosystems QuantStudio, Thermo Fisher Scientific, USA) and a SYBR Green Master Mix (PowerUp SYBR Green Master Mix, Applied Biosystems, USA). The relative mRNA expression levels were calculated using the 2^−ΔΔCt^ method. The Ct (threshold cycle) values of the target genes were normalized to the Ct values of the housekeeping gene. The fold change in expression levels relative to the control group was determined for each target gene. Primers were designed and synthesized by Shanghai BioTNT Co., Ltd., and the sequences are presented in Table S1.

### 2.27 Enzyme‐Linked Immunosorbent Assay

Cytokine abundance of IL1B (Cat. No. 88–7013A‐88, ThermoFisher Scientific, China), IL18 (Cat. No. ab216165, abcam, UK), and TNF‐α (Cat. No. ab208348, abcam, UK) was measured by enzyme‐linked immunosorbent assay (ELISA) according to the manufacturer's instructions.

### 2.28 Behavioral Assessment

All behavioral assessments and animal procedures were conducted in strict adherence to ARRIVE guidelines and were approved by IACUC of ZICLA (Approval No. ZJCLA‐IACUC‐20050101). The open field test (OFT) was conducted to assess pain‐associated behavior in mice [26, 27]. The apparatus consisted of a square arena (50 × 50 × 50 cm) constructed from opaque, non‐reflective gray polyvinyl chloride (PVC) to provide optimal contrast for video tracking. The arena was uniformly illuminated from above to a consistent level of 150 lux. Prior to testing, mice were habituated to the testing room for at least 60 min. For each trial, an individual mouse was gently placed into the center of the arena, and its spontaneous behavior was recorded for a 5‐min session by an overhead digital camera linked to an automated video‐tracking system. Between subjects, the apparatus was thoroughly cleaned with 70% ethanol and dried completely to eliminate residual olfactory cues.

Nociceptive sensitivity was evaluated by quantifying mechanical allodynia and thermal hyperalgesia. Prior to each testing session, mice were habituated to the experimental apparatus and environment for at least 30 min. Mechanical sensitivity was assessed by measuring the paw withdrawal threshold (PWT) using von Frey filaments (Stoelting Co., IL, USA). Briefly, animals were placed on an elevated wire mesh grid, and calibrated filaments were applied perpendicularly to the mid‐plantar surface of the hind paw with sufficient force to cause slight buckling. A positive response was defined as a brisk withdrawal or flinching of the paw upon stimulation. After 30 min of PWT assessment, thermal hyperalgesia was measured by determining the paw withdrawal latency (PWL). Mice were placed on a temperature‐controlled plate (50°C), and the latency (in seconds) to elicit a paw withdrawal response was recorded. A 20‐s cutoff was imposed to prevent tissue damage. Three trials were conducted for the left hind paw in each mouse, with a minimum interval of 5 min between stimuli, and the average latency was calculated.

The tail flick test was utilized to evaluate nociceptive reflexes in response to a thermal stimulus in the tail [28, 29]. Detailed assessment was performed using a high‐powered laser (1 W, 1mm^2^, 730 nm) beam, which focused on the dorsal surface of the tail. Mice were gently restrained in a transparent acrylic immobilizer, ensuring the tail remained free. The latency period, defined as the time elapsed between the onset of the heat stimulus and the abrupt withdrawal of the tail, was recorded via a timer. To prevent tissue damage or thermal hyperalgesia, a strict cut‐off time of 40 s was enforced. To minimize observer bias, all behavioral assessments were conducted by investigators blinded to the specific treatment allocation of each animal.

### 2.29 Statistical Analysis

All quantitative experiments were conducted with a minimum of three independent biological replicates. Pre‐processing of data involved the evaluation of outliers using the ROUT method (Q = 1%); no data transformation was performed. Data are presented as the mean ± standard deviation (SD). The exact sample size (n) for each statistical analysis is explicitly stated in the respective figure legends. Prior to analysis, all datasets were assessed for normality using the Shapiro–Wilk test and for homogeneity of variances using the Brown–Forsythe test. For comparisons between two independent groups, an unpaired, two‐tailed Student's *t*‐test was used. For comparisons involving three or more groups with a single independent variable, a one‐way analysis of variance (ANOVA) was performed. For time‐course or longitudinal data involving multiple groups evaluated across different time points, a two‐way ANOVA was utilized. Significant ANOVA results were followed by Tukey's HSD (Honest Significant Difference) post hoc test for multiple pairwise comparisons. All statistical analyses were performed using GraphPad Prism (Version 10, GraphPad Software, San Diego, CA). Differences were considered statistically significant at a two‐sided *p*‐value of less than 0.05. Statistical significance is represented graphically as *p < 0.05, **p < 0.01, ***p < 0.001, and ****p < 0.0001, with specific symbols “#” defined in the respective figure legends.

## 3 Results

### 3.1 Radiological, Histological, and Single‐Cell Transcriptional Analysis of IVDD and OA

**FIGURE 1:**
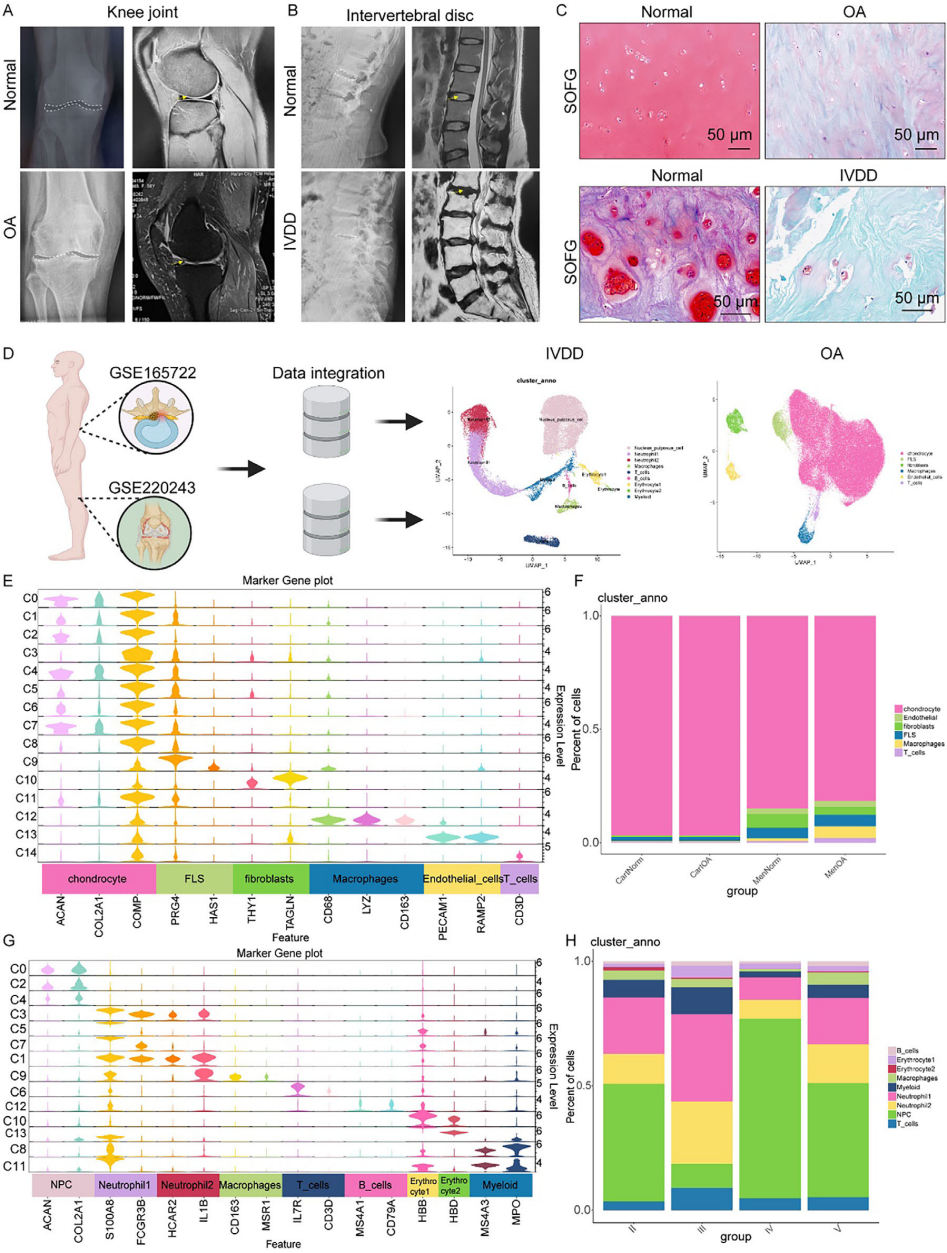
Shared pathological hallmarks and cellular ecosystems in OA and IVDD. (A) Representative radiographs and MRI scans of normal and osteoarthritic human knee joints. Radiographs of the OA knee show narrowed joint space, while T2‐weighted MRI reveals thinning and roughening of articular cartilage (yellow arrowhead). (B) Representative radiographs and MRI scans of healthy and degenerated human intervertebral discs. Radiographs of the IVDD spine demonstrate decreased disc height, with corresponding T2‐weighted MRI showing reduced signal intensity (yellow arrowhead), indicative of dehydration. (C) Representative SOFG histological staining of human knee cartilage and intervertebral disc tissue, illustrating proteoglycan loss, disorganized ECM, and chondrocyte clustering in OA and IVDD. Scale bar, 50 µm. (D) Integrated UMAP visualization of single‐cell transcriptomes from knee joint (GSE220243) and intervertebral disc (GSE165722) datasets. (E) The expression levels of canonical marker genes used to identify the primary cell types in the OA dataset. (F) Bar plot illustrating the proportional changes in major cell populations between control and OA tissues. (G) The expression levels of key marker genes for the identified cell populations in the IVDD dataset. (H) Bar plot showing the relative abundance of major cell types in different degenerative degrees (grade II to grade V).

A comparative analysis of normal and pathological states in the human knee joint and intervertebral disc was conducted using imaging and histology. Radiographs and MRI of a normal knee display a well‐maintained joint space and smooth cartilage (Figure 1A). Radiographs reveal a narrowed joint space, while MRI shows rough, thin cartilage (yellow arrowhead) in OA (Figure 1A). For the intervertebral discs, radiographs of healthy discs show normal height, with MRI displaying homogenous signal (Figure 1B). In IVDD, radiographs reveal decreased disc height, and MRI shows reduced signal intensity (yellow arrowhead), indicating dehydration (Figure 1B). Histological assessment of SOFG revealed disrupted ECM, reduced proteoglycans, and chondrocyte clustering in both OA and IVDD tissues. These findings highlight structural similarities and changes in OA and IVDD progression.

To define the distinct cellular ecosystems and identify transcriptomic signatures associated with the pathology of OA and IVDD, we integrated two publicly available single‐cell RNA sequencing (scRNA‐seq) datasets, GSE220243 (knee joint) and GSE165722 (intervertebral disc) (Figure 1D). Following data integration and rigorous quality control, unsupervised clustering projected via UMAP revealed cellular populations for OA and IVDD tissues. In the OA dataset, a total of 7 primary cell types were identified, including chondrocytes, fibroblast‐like synoviocytes (FLS), fibroblasts, macrophages, endothelial cells, and T cells (Figure 1D,E). Chondrocytes were characterized by high expression of ACAN, COL2A1, and COMP; FLS were defined by robust expression of PRG4 and HAS1; macrophages were confirmed by high expression of CD68, LYZ, and CD163 (Figure 1E). Similarly, in the IVDD dataset, the analysis resolved 9 distinct cell populations (Figure 1D,G), comprising NPCs, Neutrophil1, Neutrophil2, macrophages, B cells, T cells, Erythrocyte1, Erythrocyte2, and myeloid cells (Figure 1H). Cell annotation was validated using specific marker expression profiles (Figure 1G). For example, NPCs were identified by high expression of ACAN and COL2A1; macrophages were confirmed by CD163 and MSR1 (Figure 1G). To provide a comprehensive landscape of transcriptional alterations, we visualized the differentially expressed genes across all major cell types in IVDD and OA. In IVDD, NPCs exhibited upregulation of fibrotic markers like CTGF and DCN, while macrophages showed increased expression of chemokines and antigen presentation such as CD74, HLA‐DRA, CCL3, and CXCL3 (Figure S1A). In the OA microenvironment, macrophages in OA exhibited significant upregulation of genes involved in antigen presentation and chemokines, such as HLA‐DRA, HLA‐DRB1, CD74, CXCL8, and RNASE1 (Figure S1B). Additionally, compositional analysis demonstrated notable shifts in the relative abundance of immune cell populations, especially macrophages, in both OA and IVDD tissues, highlighting a prominent inflammatory microenvironment in these degenerative conditions (Figure 1F,H). These global profiles underscore the similar transcriptional reprogramming that occurs during tissue degeneration in intervertebral discs and the knee joint.

### 3.2 Single‐Cell Sequencing Analysis of NPCs and Chondrocyte Subpopulations in IVDD and OA

To further dissect the cellular heterogeneity within the NPC population during IVDD, we performed subclustering analysis on the identified NPCs. Unsupervised clustering revealed eight distinct subpopulations, designated NPC0 through NPC7 (Figure S2A). Compositional analysis demonstrated a significant shift in the relative proportions of these subpopulations between control and IVDD, with NPC2, NPC3, and NPC7 remarkably decreased, and NPC5 increased in the severely degenerated IVDs, indicating a dynamic cellular response to the degenerative microenvironment (Figure S2B). Volcano plots of DEGs highlighted unique transcriptomic signatures for each subcluster. NPC0 exhibited high expression of matrix genes such as COL2A1 and ACAN, whereas NPC4 presented elevated MMP3 and lowered COL2A1, and NPC3 showed raised COL1A1 levels (Figure S2C). To model the progression of NPC states, pseudotime trajectory analysis was performed, which revealed a clear progression, with clusters like NPC0 and NPC2 positioned at the beginning and clusters such as NPC5 located at the terminal end, suggesting a path of cellular state transition during disease progression (Figure S2D). A heatmap of gene expression dynamics along this pseudotime trajectory visualized distinct modules of gene regulation, including an early‐stage module enriched for homeostatic matrix proteins (VCAN, PRG4) and a late‐stage module characterized by fibrotic and catabolic markers (MMP13, COL1A2, ASPN) (Figure S2E).

A parallel subclustering analysis was conducted on chondrocytes from the OA dataset to characterize their heterogeneity. This analysis identified eight distinct chondrocyte subpopulations (Chon0‐Chon7) (Figure S3A). The proportional distribution of some subclusters was remarkably altered in the OA group compared to controls, mirroring the cellular shifts (Figure S3B). Each subcluster displayed a unique gene expression profile, as illustrated by volcano plots. Chon0 was marked by low expression of IGFBP5 and COL1A1, whereas Chon3 presented raised COL1A1 expression and Chon5 showed raised MMP3 levels (Figure S3C). Pseudotime analysis of the chondrocyte population delineated a pathological trajectory from a healthy to a degenerative state, showing that Chon3 and Chon6 were localized at the degenerative state (Figure S3D,E). Gene expression dynamics along this trajectory were visualized, revealing a progressive downregulation of cartilage matrix genes including COL2A1 and ACAN and a concomitant upregulation of genes associated with hypertrophy, inflammation, and matrix degradation, including INHBA, S100A family members, and COL3A1 (Figure S3F).

To identify common molecular mechanisms driving degeneration in OA and IVDD, we compared the transcriptomic profiles of terminal‐stage cell subpopulations. A Venn diagram analysis of DEGs between the OA‐specific Chon5 subcluster and the IVDD‐specific NPC4 subcluster revealed an overlap of 107 genes, including key regulators of senescence, stress response, and inflammation such as CDKN1A, SOD2, NFKBIA, and CHI3L1 (Figure S4A). KEGG pathway enrichment analysis of these shared DEGs identified significant enrichment in pathways critical to inflammation and catabolism, most notably the “TNF signaling pathway,” “IL‐17 signaling pathway,” and “NF‐kappa B signaling pathway” (Figure S4B). Similarly, comparison of the Chon3 and NPC3 subclusters identified 89 common DEGs, including pro‐fibrotic and matrix‐remodeling genes like S100A4, DKK3, COL1A1, and TNC (Figure S4D). Pathway analysis of this shared gene set highlighted enrichment for “Focal adhesion,” “PI3K‐Akt signaling pathway,” and “ECM‐receptor interaction,” consistent with a fibrotic cellular phenotype (Figure S4E). Moreover, analysis of transcription factor regulons indicated that transcription factors such as NFIL3, FOSB, and REL were highly active in the terminal‐stage Chon5 and NPC4 clusters, while factors like TWIST1 and SOX9 were active in Chon3 and NPC3 clusters, suggesting shared regulatory control of these pathological cell states (Figure S4C,F).

### 3.3 Macrophage Heterogeneity and Similarity in IVDD and OA

To explore the diversity within the macrophage populations, we performed independent subclustering of macrophages from both datasets. In IVDD, this analysis resolved five distinct macrophage subpopulations (designated 0–4), with IL‐1β^+^ macrophages (Mac0) presenting elevated inflammation indicators such as IL1B, NLRP3, and CCL20 (Figure S5A,B). Similarly, subclustering of OA macrophages identified seven distinct subpopulations (designated 0–6), with IL‐1β^+^ macrophages (Mac0) showing raised levels of CCL3 and IL1B (Figure S5C,D). The presence of these diverse macrophage subsets in both diseases suggests a complex myeloid response, likely comprising cells with distinct pro‐inflammatory, anti‐inflammatory, and tissue‐remodeling functions that contribute to the overall pathological state.

Then we performed a comparative functional enrichment analysis on the IL‐1β^+^ macrophages from both IVDD and OA samples. KEGG pathway analysis revealed a striking overlap in the activated biological processes. In both conditions, IL‐1β^+^ macrophages (Mac0) were significantly enriched for pro‐inflammatory signaling cascades, including the “NF‐kappa B signaling pathway,” “NOD‐like receptor signaling pathway,” “Toll‐like receptor signaling pathway”, and “TNF signaling pathway” (Figure S6A,B), underscoring a conserved pro‐inflammatory and tissue‐destructive functional state of macrophages across both degenerative diseases.

### 3.4 Pro‐Inflammatory Macrophages Drive Pathological Crosstalk in IVDD and OA

**FIGURE 2:**
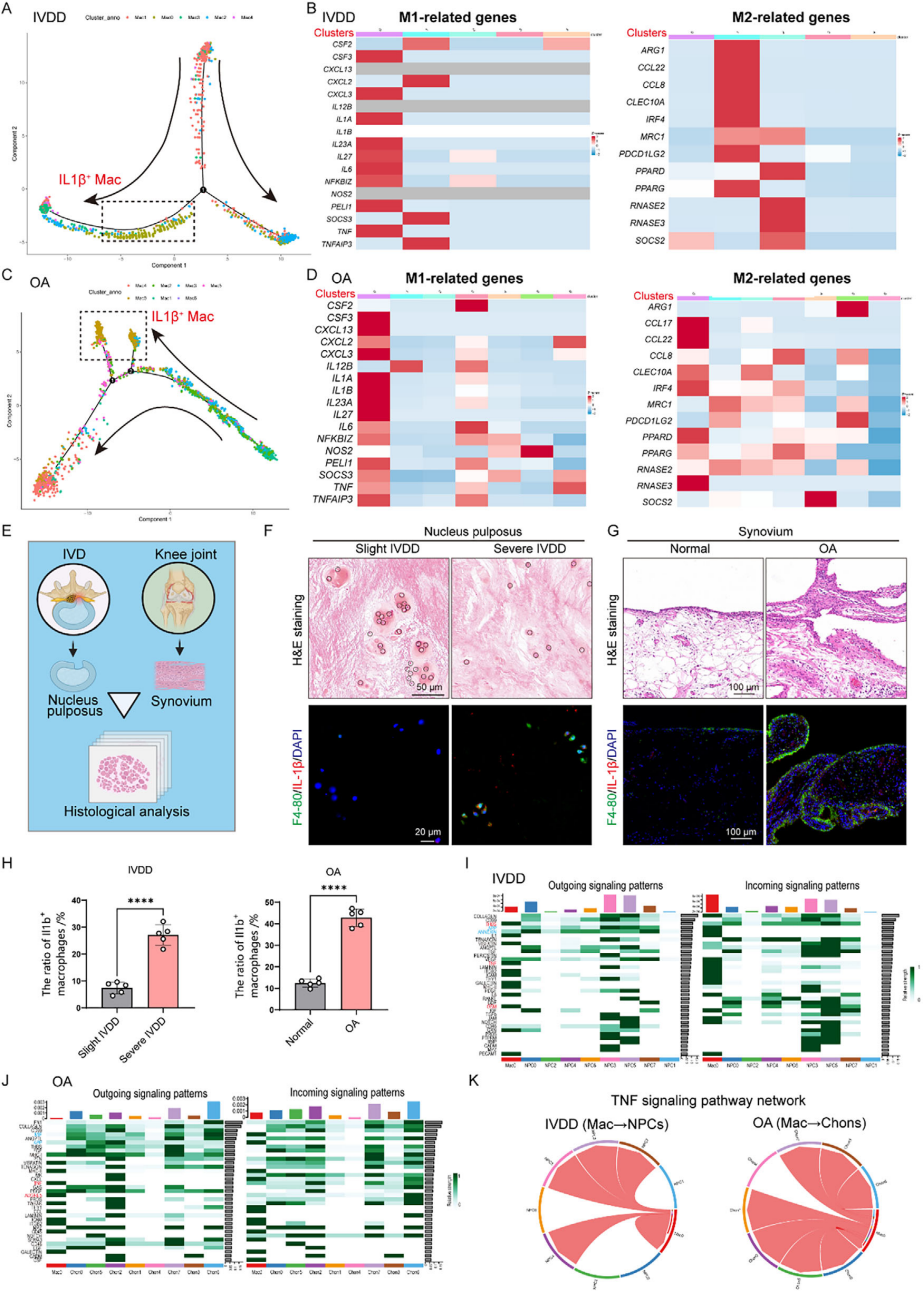
A conserved pro‐inflammatory macrophage subpopulation drives progression of IVDD and OA. (A) Pseudotime trajectory analysis of macrophage subpopulations in the IVDD dataset, illustrating their activation and differentiation paths. (B) Heatmap displaying the expression of representative pro‐inflammatory and anti‐inflammatory marker genes across macrophage subpopulations in IVDD. (C) Pseudotime trajectory analysis of macrophage subpopulations in the OA dataset. (D) Heatmap showing the expression of key pro‐inflammatory and anti‐inflammatory marker genes across OA macrophage subpopulations, confirming a potent pro‐inflammatory phenotype in IL‐1β^+^ macrophages (Mac0). (E) Schematic diagram of human histological analysis. (F) H&E staining and co‐immunofluorescence staining for the macrophage marker F4/80 and the pro‐inflammatory cytokine IL‐1β of NP tissues from mild and severe IVDD patients. Scale bar, 100 µm for H&E staining and 20 µm for immunofluorescence staining. (G) H&E staining and co‐immunofluorescence staining for the macrophage marker F4/80 and the pro‐inflammatory cytokine IL‐1β of synovium tissues from normal and OA patients. Scale bar, 100 µm for H&E staining and 100 µm for immunofluorescence staining. (H) Quantification analysis of the F4/80^+^IL‐1β^+^ macrophages. (I) Heatmaps visualizing the outgoing and incoming signaling patterns across Mac0 and NPC0‐7 subpopulations in the IVDD microenvironment. (J) Heatmaps of outgoing and incoming signaling patterns in the OA microenvironment, highlighting Mac0 as a major signaling hub targeting pathological chondrocyte subpopulations. (K) Chord diagrams of the TNF signaling pathway network in IVDD and OA, demonstrating that the inflammatory Mac0 subpopulation is the predominant source of TNF, which specifically targets degenerative resident cells in both IVDD and OA. Data are presented as mean ± SD. For comparisons between two groups, an unpaired, two‐tailed Student's *t*‐test was used. The comparisons between multiple groups were conducted using one‐way ANOVA followed by Tukey's post hoc test. *, *p* < 0.05; **, *p* < 0.01; ***, *p* < 0.001; ****, *p* < 0.0001; ns = not significant.

To dissect the functional states of macrophage subpopulations, we performed pseudotime trajectory analysis. This revealed distinct activation trajectories for macrophages in both IVDD and OA (Figure 2A,C), suggesting a dynamic progression from a precursor state to multiple terminally differentiated states. To characterize the functional polarization of these subpopulations, we examined the expression of pro‐inflammatory and anti‐inflammatory marker genes. In IVDD, the Mac0 and Mac1 subpopulations exhibited robust expression of pro‐inflammatory M1‐associated genes, including TNF, IL1A, IL1B, and NFKBIZ (Figure 2B). Interestingly, the Mac1 subpopulation also co‐expressed several anti‐inflammatory‐related genes such as ARG1, CCL22, and MRC1, indicating a mixed‐phenotype state. Similarly, in OA, the Mac0 subpopulation displayed a strong pro‐inflammatory signature, with high expression of IL1B, IL6, and NFKBIZ, whereas the anti‐inflammatory genes such as ARG1 and MRC1 were lowered in Mac0 (Figure 2D). These findings highlight that specific macrophage subsets, particularly the Mac0 cluster in both diseases, adopt a potent pro‐inflammatory phenotype.

To explicitly bridge our single‐cell transcriptomic findings with in situ spatial pathology, we conducted histological and immunofluorescence analyses on NP tissues from IVDD patients and synovial tissues from OA patients (Figure 2E). Crucially, to ensure our cellular data accurately reflected meaningful pathology and to avoid sampling arbitrary regions, our histological observations and subsequent fluorescence imaging were deliberately focused on the anatomical epicenters of tissue destruction. H&E staining of severe IVDD NP tissue revealed profound matrix structural collapse, cleft formation, and fibrotic replacement characteristics of advanced disc degeneration, while OA synovium showed marked synovial hyperplasia and inflammatory cell infiltration into the subintimal stroma compared to their respective controls (Figure 2F,G). To confirm the inflammatory nature and spatial distribution of the macrophages within these specific lesions, we performed co‐immunofluorescence staining for the macrophage marker F4/80 and the key pro‐inflammatory cytokine IL‐1β. This analysis revealed a significant increase in the population of IL‐1β‐expressing macrophages (F4/80^+^IL‐1β^+^) directly localized within the epicenter of the pathological microenvironments (adjacent to structural fissures in IVDD and within the hyperplastic synovial lining in OA), providing in situ confirmation of our single‐cell transcriptomic findings (Figure 2F–H).

Given the prominent pro‐inflammatory state of macrophages and their direct localization at the sites of tissue destruction, we next investigated their role in mediating intercellular communication. We performed cell–cell interaction analysis, which identified the Mac0 subpopulation as a major signaling hub in both the IVDD and OA microenvironments. In both IVDD and OA, the outgoing signaling patterns showed that Mac0 was a primary source of numerous pro‐inflammatory and matrix‐degrading ligands, while the incoming patterns revealed that pathological NPC, such as NPC3 and NPC5, and chondrocyte such as Chon2, Chon6, Chon7 subpopulations were the principal recipients of these signals (Figure 2I,J). Chord diagrams demonstrated that in both IVDD and OA, the inflammatory Mac0 subpopulation was the predominant source of TNF signaling, which specifically targeted the degenerative NPCs and chondrocytes (Figure 2K). To ascertain the most biologically consequential communication networks driving the observed lesions, we filtered the ligand–receptor interactions based on calculated interaction probabilities. Importantly, the selection of the TNF‐TNFRSF1A signaling axis for detailed highlighting was not arbitrary. Unbiased computational ranking via the CellChat algorithm identified this specific ligand–receptor pair as possessing the highest mathematical communication probability and interaction strength between Mac0 macrophages and the terminally differentiated resident cells (NPCs/Chondrocytes). This quantitative computational prediction explains the mechanism of apoptosis and matrix catabolism driving the severe structural clefts and hyperplastic lesions observed in our spatial histological analysis. The specific ligand‐receptor pair analysis showed that TNF and its receptor TNFRSF1A were the predominant contributors to the overall TNF signaling strength, which plays an important role in pro‐inflammatory macrophages in both IVDD and OA (Figure S7). Collectively, these results underscore a conserved pathological mechanism wherein pro‐inflammatory macrophages physically infiltrate the lesion sites and drive tissue degeneration through targeted catabolic signaling to resident structural cells in both IVDD and OA.

### 3.5 Screening of Potent Pyroptosis and NF‐κB Pathway Inhibitor

**FIGURE 3:**
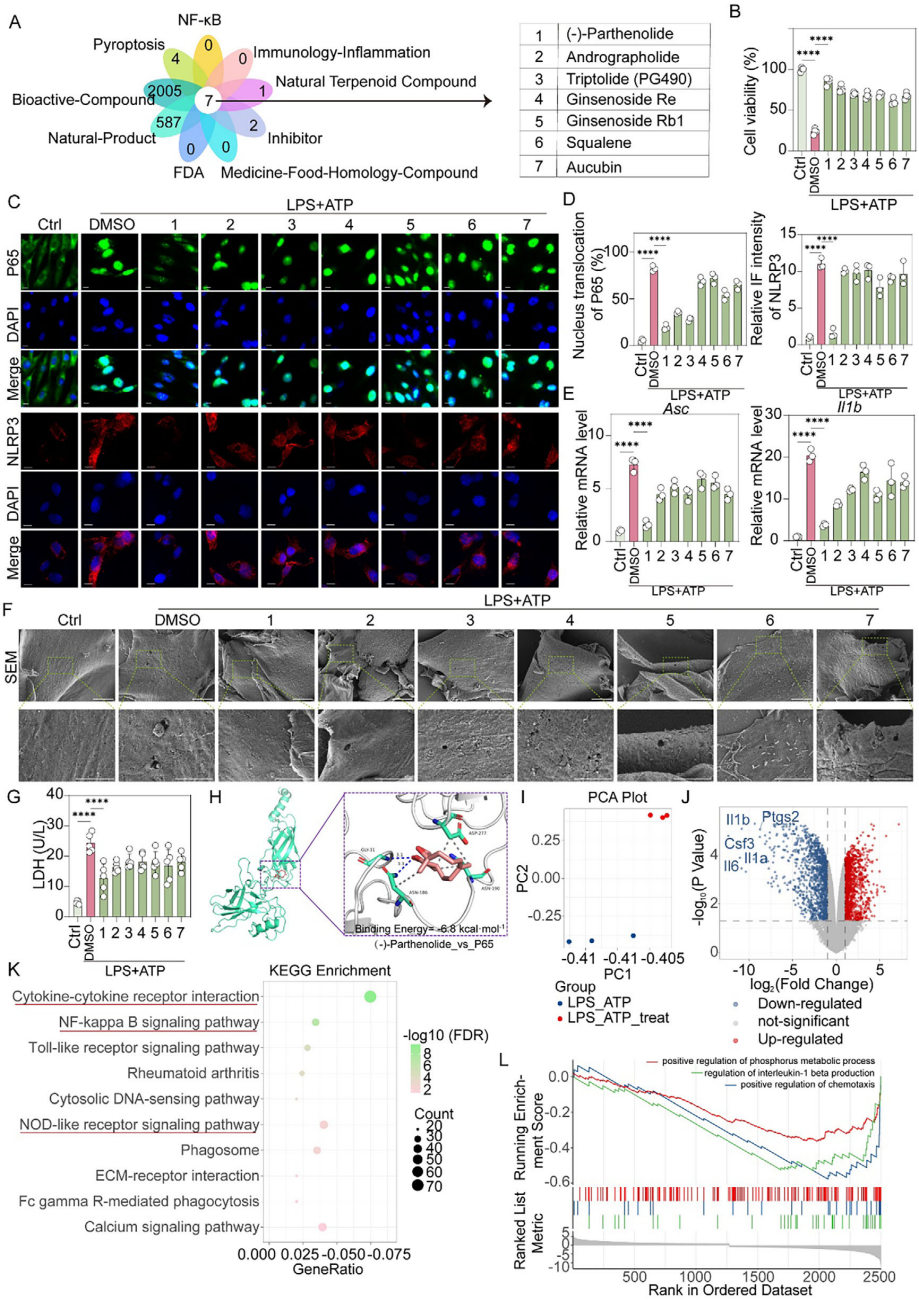
PTL is identified as a potent inhibitor of LPS+ATP‐induced pyroptosis and NF‐κB activation in macrophages. (A) Venn diagram illustrating the screening of seven candidate natural terpenoid compounds from nine databases. (B) Cell viability of LPS+ATP‐stimulated BMDMs treated with the seven candidate compounds (n = 3). (C) Representative IF staining images for P65 nuclear translocation and NLRP3 expression in BMDMs from different treatment groups (n = 3). Scale bar, 20 µm. (D) Quantitative analysis of P65 nuclear translocation and NLRP3 fluorescence intensity (n = 3). (E) RT‐qPCR analysis of pyroptosis‐related genes *Asc* and *Il1b* (n = 3). (F) Representative SEM images showing pyroptotic pores on the cell membrane (n = 3). The bottom panels present high‐magnification views of the regions outlined by the dashed boxes in the top panels. Scale bars, 10 µm (top images) and 5 µm (bottom images). (G) Quantification of LDH release in the supernatant of treated BMDMs (n = 5). (H) Molecular docking model illustrating the binding interaction between PTL and the P65 protein. (I) PCA of transcriptomic data from macrophages (n = 3). (J) Volcano plot of DEGs comparing the LPS+ATP‐stimulated cells with or without PTL treatment (n = 3). (K) KEGG pathway enrichment analysis of DEGs. (L) GSEA showing inhibition of chemotaxis and interleukin‐1 beta production pathways by PTL. Data are presented as mean ± SD. The comparisons between multiple groups were conducted using one‐way ANOVA followed by Tukey's post hoc test. *p < 0.05; **p < 0.01; ***p < 0.001; ****p < 0.0001; ns = not significant.

Based on the above findings showing that NF‐κB, NOD‐like receptor signaling pathway, and TNF signaling pathway were significantly enriched in both IVDD and OA, we speculated that the inflammation signaling and inflammatory cell dysfunction like pyroptosis play some role in promoting degeneration. Thus, we have made an attempt to find natural anti‐inflammatory and anti‐pyroptosis compounds. To ensure a rigorous and targeted selection process, we established strict screening criteria aimed at identifying molecules that were structurally stable, biologically safe, and theoretically active against our specific target pathways. The intersection of compounds from nine databases was taken, including “Pyroptosis,” “NF‐kappa B,” “Immunology‐Inflammation”, “Natural Terpenoid Compound,” “Inhibitor,” “Medicine‐Food‐Homology‐Compound,” “FDA Approved compound,” “Natural‐Product,” and “Bioactive‐Compound.” Seven candidate natural terpenoid compounds were obtained for further investigation, including (−)‐Parthenolide, Andrographolide, Triptolide, Ginsenoside Re, Ginsenoside Rb1, Squalene, and Aucubin (Figure 3A). For in vitro validation, BMDMs were primed with LPS (1 µg/mL) for 4 h, followed by ATP (5 mM) stimulation for 1 h to induce canonical pyroptosis. Subsequent in vitro validation was performed using BMDMs stimulated with LPS+ATP to induce an inflammatory pyroptosis model. A cell viability assay revealed that all seven compounds (10 µM for 24 h) offered protection, with (−)‐Parthenolide (PTL, compound 1) demonstrating the most pronounced restorative effect (Figure 3B). Furthermore, we investigated the effect of these compounds on the NF‐κB pathway. IF staining for the P65 subunit showed marked nuclear translocation in the LPS+ATP‐stimulated group, indicative of NF‐κB activation (Figure 3C). Treatment with PTL effectively inhibited P65 nuclear translocation, an effect superior to the other tested compounds, which exhibited only marginal or inconsistent suppression of this cascade under severe inflammatory stress (Figure 3C,D). Besides, IF staining showed that PTL resulted in superiorly remarkable decrease in the NLRP3 IF intensity (Figure 3C,D). Notably, RT‐qPCR analysis showed that PTL treatment led to the most significant downregulation of the pyroptosis‐related genes, *Asc* and *Il1b* (Figure 3E). Morphological evaluation via SEM provided direct visual evidence of the anti‐pyroptotic mechanism. While the control macrophages exhibited continuous and intact cell membranes, cells in the LPS+ATP‐stimulated DMSO group displayed the classic, specific ultrastructural hallmarks of gasdermin‐mediated pyroptosis, characterized by the widespread formation of distinct, discrete membrane pores (Figure 3F). These pores act as physical conduits for the leakage of intracellular contents and inflammatory mediators. Treatment with PTL effectively preserved overall membrane continuity and essentially abolished the formation of these characteristic pyroptotic pores. Consistent with this structural preservation, the LDH release assay showed that PTL led to the most significant downregulation of LDH levels (Figure 3G). Furthermore, molecular docking simulations indicated a high binding affinity between PTL and the P65 protein. A binding energy of −6.8 kcal/mol was calculated, which is well below the threshold of −5 kcal/mol typically considered indicative of a stable and spontaneous interaction (Figure 3H).

To elucidate the molecular mechanisms of the treatment, we performed transcriptomic analysis on macrophages subjected to LPS+ATP stimuli with or without PTL treatment. PCA and hierarchical clustering revealed a distinct transcriptomic profile for the treated group, indicating a significant and consistent global shift in gene expression (Figure 3I and Figure S8A). Volcano plot analysis identified numerous DEGs, with significant downregulation of key pro‐inflammatory mediators including *Il1b*, *Ptgs2*, *Il1a*, and *Il6* following PTL treatment (Figure 3J). KEGG analysis revealed that the inflammatory response was predominantly characterized by the enrichment of key signaling cascades, including the cytokine–cytokine receptor interaction, NF‐kappa B, Toll‐like receptor, and NOD‐like receptor signaling pathways (Figure 3K). Furthermore, GSEA analysis showed remarkable inhibition of pathways including positive regulation of chemotaxis and the regulation of interleukin‐1 beta production in the PTL‐treated groups (Figure 3L). GO enrichment analysis of the DEGs confirmed a profound enrichment of inflammatory pathways, underscoring the PTL's potent anti‐inflammatory effect (Figure S8B,C). Our above findings confirmed that PTL plays a pivotal role in modulating the inflammatory response via the NF‐kappa B pathway. Based on these comprehensive screening results, we justified focusing on PTL as the core pharmacological payload for our final nanoparticle design. While the other six candidates satisfied theoretical in silico criteria, only PTL provided the robust, multilevel intracellular blockade of the NF‐κB and pyroptosis axes required to arrest degeneration. Furthermore, as a hydrophobic natural compound with traditionally poor in vivo bioavailability, PTL profoundly benefits from nanoparticle encapsulation. This design establishes a highly synergistic therapeutic mechanism: the biomimetic nanoparticle shell acts extracellularly to neutralize upstream inflammatory ligands via decoy receptors, while the core reliably delivers PTL to silence downstream intracellular inflammatory cascades.

### 3.6 Engineering of a Pro‐Inflammatory Macrophage‐Biomimetic Nanosystem

**FIGURE 4:**
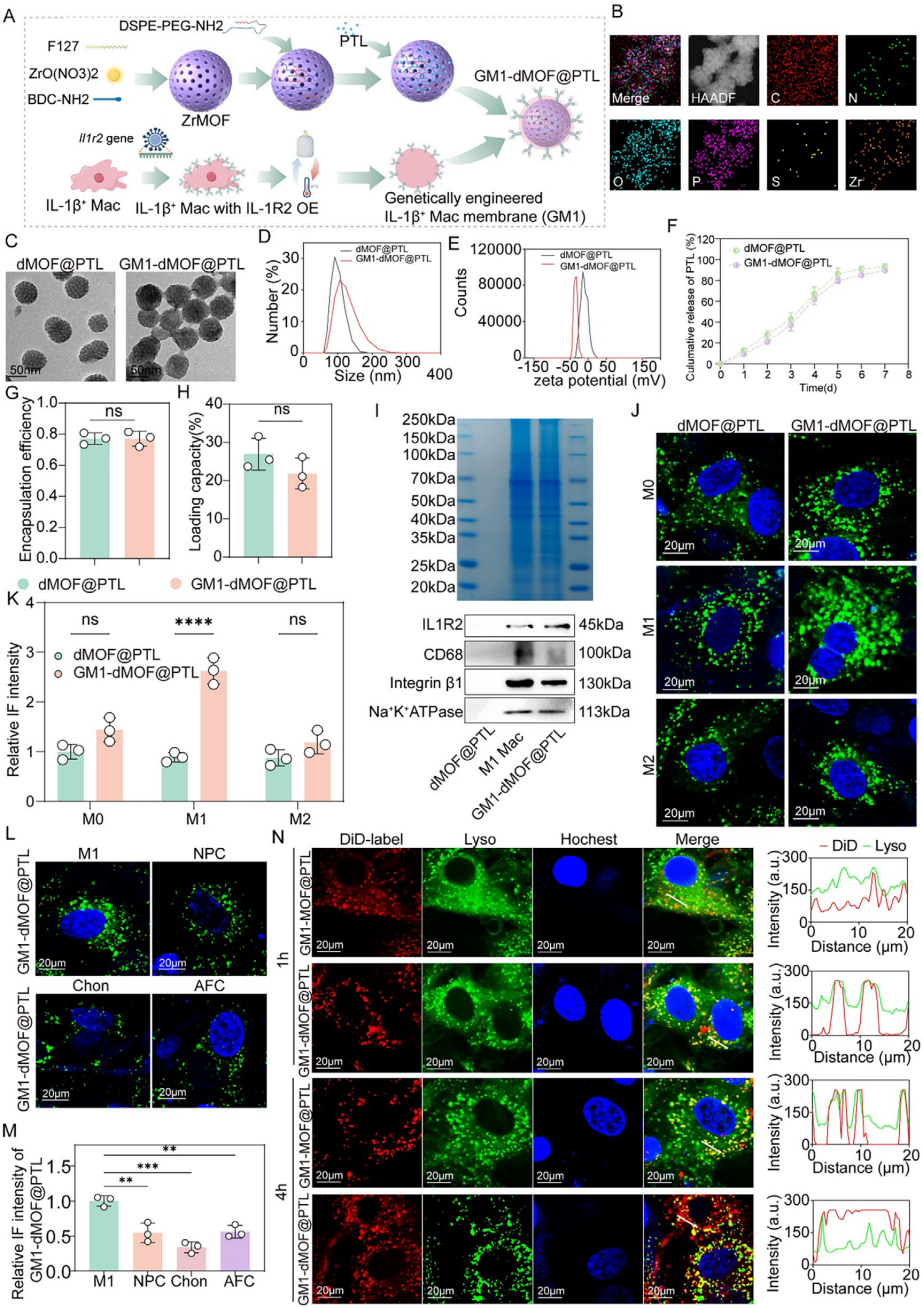
Fabrication, characterization, and targeted uptake of the M1 macrophage‐biomimetic nanosystem GM1‐dMOF@PTL. (A) Schematic illustration of the synthesis of the genetically engineered inflammatory macrophage membrane‐camouflaged, PTL‐loaded MOF nanosystem. (B) HAADF imaging and corresponding elemental mapping of C, N, O, P, S, and Zr in GM1‐dMOF@PTL nanoparticles. (C) Representative TEM images of uncoated dMOF@PTL and M1 macrophage membrane‐camouflaged GM1‐dMOF@PTL. Scale bar, 50 nm. (D, E) Hydrodynamic diameter distribution and zeta potential of nanoparticles before and after membrane coating (n = 3). (F) In vitro drug release profiles of PTL from dMOF@PTL and GM1‐dMOF@PTL over 7 days (n = 3). (G–H) Encapsulation efficiency and drug loading capacity of PTL in the nanoformulations (n = 3). (I) SDS‐PAGE and Western blot analysis of proteins from dMOF@PTL, inflammatory M1 macrophage, and GM1‐dMOF@PTL, confirming the successful transfer of key inflammatory macrophage membrane proteins (CD68, Integrin β1, Na^+^K^+^ATPase) and the overexpression of IL1R2. The full‐length, uncropped western blot images corresponding to the immunoblots presented in this figure are available in the Supporting Information of western blot images. (J, K) Representative fluorescence images and quantitative analysis demonstrating the homologous targeting and enhanced uptake of GM1‐dMOF@PTL by pro‐inflammatory M1 macrophages compared to M0 and anti‐inflammatory M2 macrophages (n = 3). Scale bar, 20 µm. (L, M) Representative images and quantitative analysis showing the specific targeting of GM1‐dMOF@PTL to inflammatory M1 macrophages over NPCs and chondrocytes (n = 3). Scale bar, 20 µm. (N) Confocal microscopy images tracking the intracellular trafficking of DiD‐labeled GM1‐dMOF@PTL in pro‐inflammatory macrophages over 24 h. Lysosomes were stained with LysoTracker. Data are presented as mean ± SD. The comparisons between multiple groups were conducted using one‐way ANOVA followed by Tukey's post hoc test. The comparisons between two groups were conducted using an unpaired, two‐tailed Student's *t*‐test. *p < 0.05, **p < 0.01, ***p < 0.001, ****p < 0.0001; ns = not significant.

Despite PTL's potent anti‐inflammatory effects, its clinical translation for IVDD and OA is impeded by significant hurdles. PTL's unfavorable physicochemical profile, including poor aqueous solubility, limits its bioavailability. Furthermore, direct local administration into avascular IVD or joint tissues is plagued by rapid clearance, yielding only a transient therapeutic window and necessitating frequent, invasive injections. Critically, nonspecific delivery fails to target the primary pathogenic drivers, pro‐inflammatory M1 macrophages, risking off‐target cytotoxicity to homeostatic cells. To surmount these multifaceted challenges, we rationally engineered a novel biomimetic nanosystem. Our strategy involved creating a decoy‐receptor‐functionalized nanosystem for targeted delivery to pro‐inflammatory macrophages. To this end, we genetically engineered pro‐inflammatory macrophages to overexpress the Interleukin‐1 receptor type 2 (*Il1r2*), a decoy receptor that binds pro‐inflammatory IL‐1β without transducing a signal. These GM1 cell membranes were then used to camouflage a multifunctional core nanoparticle, which consisted of a PTL‐loaded Zirconium‐based metal–organic framework (ZrMOF) surface‐modified with DSPE‐PEG‐NH_2_ (“d”) to facilitate lysosomal escape. This process yielded the final biomimetic nanosystem, GM1‐dMOF@PTL (Figure 4A).

The successful fabrication of this core–shell nanostructure was rigorously validated. High‐angle annular dark‐field (HAADF) imaging coupled with elemental mapping confirmed the uniform coating of the GM1 membrane onto the ZrMOF core, evidenced by the colocalization of membrane‐specific elements, phosphorus (P) and sulfur (S), with the core element, zirconium (Zr) (Figure 4B). This core–shell morphology was further visualized by transmission electron microscopy (TEM), which revealed a distinct membrane layer for the GM1‐dMOF@PTL nanoparticles, a feature absent in the uncoated decoy dMOF@PTL counterparts (Figure 4C). Dynamic light scattering (DLS) analysis indicated that the hydrodynamic diameter of dMOF@PTL was approximately 97 nm, which increased to around 119 nm upon membrane coating to form GM1‐dMOF@PTL, with a concomitant shift in the surface zeta potential, confirming successful surface modification (Figure 4D,E). Crucially, as chronic degenerative joint and disc diseases clinically necessitate prolonged, sustained therapeutic interventions rather than rapid, transient drug bursts, we comprehensively evaluated the structural stability and drug release kinetics of the ZrMOF core. Zirconium‐based MOFs were specifically selected as the carrier because their robust coordination bonds between high‐valence Zr^4^^+^ clusters and carboxylate linkers confer exceptional chemical stability. TEM observation intuitively validated this ultrastable design. Following prolonged continuous incubation for up to 7 days in both a standard physiological buffer (pH 7.4) and a simulated harsh acidic inflammatory microenvironment (pH 6.5 supplemented with IL‐1β), GM1‐dMOF@PTL exhibited no signs of framework collapse or degradation. Instead, they robustly maintained their distinct spherical morphology, intact boundaries, and dense core architecture regardless of the microenvironmental stress (Figure S9A). At physiological pH (7.4), GM1‐dMOF@PTL exhibited a drug release profile similar to that of the uncoated dMOF@PTL, but with a slightly slower release rate due to the cell membrane coating. Both nanoparticles demonstrated excellent sustained‐release properties, releasing nearly 90% of the loaded drug within 7 days. Notably, after day 5, the release rate slowed down significantly, with minimal subsequent release (Figure 4F). Consistent with the remarkable structural integrity, the drug release profile was characterized by a stable, diffusion‐driven mechanism rather than carrier breakdown. In both physiological (pH 7.4) and simulated pathological (pH 6.5 + IL‐1β) conditions, GM1‐dMOF@PTL steadily released its payload over the course of 7 days, reaching a cumulative release of approximately 85%–90% (Figure S9B). This robust stability ensures that the therapeutic payload is protected during local retention and prior to cellular uptake and provides a continuous, long‐acting intracellular suppression of inflammatory pathways once successfully internalized by pathogenic macrophages at the local lesions. The encapsulation efficiency and loading capacity for PTL were determined to be approximately 80% and 20%, respectively, with no significant difference observed between the coated and uncoated formulations (Figure 4G,H). RT‐qPCR and Western blot analysis revealed that LV‐*Il1r2* resulted in remarkable overexpression of IL1R2 (Figure S9C,D). SDS‐PAGE and Western blot analyses of the nanoparticle lysates revealed that GM1‐dMOF@PTL exhibited a protein profile similar to that of the pro‐inflammatory M1 macrophage, which was absent in the uncoated dMOF@PTL (Figure 4I). The presence of key GM1 membrane proteins, including CD68, Integrin β1, and Na^+^/K+‐ATPase, on the GM1‐dMOF@PTL surface was confirmed, underscoring the successful biomimetic functionalization (Figure 4I and Figure S9E). To rigorously validate the efficiency and integrity of this biomimetic camouflage, a quantitative densitometric analysis was performed to compare the specific protein densities on the GM1‐dMOF@PTL versus the native macrophage source. This quantitative evaluation objectively revealed that while there was a slight reduction in the density of integral membrane markers such as CD68 and Integrin β1, a universally recognized phenomenon attributed to the mechanical shear forces generated during the vesicle extrusion process, the native protein landscape was successfully conserved on the GM1‐dMOF@PTL surface (Figure S9E). Notably, this level of protein retention remains highly abundant and is entirely sufficient to confer the required homotypic targeting capabilities, as subsequently verified in our cellular uptake assays. Most importantly, the quantitative analysis rigorously reaffirmed that the core therapeutic target, the engineered IL‐1R2 decoy receptor, was substantially and significantly enriched on the surface of GM1‐dMOF@PTL compared to the unmodified native macrophage membranes, fulfilling the decoy design rationale (Figure 4I and Figure S9E). We then assessed the IL‐1β‐blocking‐induced therapeutic potential of the nanosystem in vitro. In an IL‐1β‐induced inflammatory model of macrophages, both GM1‐dMOF@PTL and dMOF@PTL significantly downregulated the mRNA expression of key pro‐inflammatory cytokines, including *Il1b*, *Il6*, *Ifng*, *Tnf*, and *Il18* (Figure S9F–J). Notably, GM1‐dMOF@PTL demonstrated a significantly more potent anti‐inflammatory effect than its uncoated counterpart across all tested cytokines, underscoring the therapeutic benefit of the engineered targeting strategy (Figure S9F–J).

This enhanced efficacy was attributed to superior cellular targeting. We evaluated the uptake of fluorescently labeled nanoparticles by macrophages with different phenotypes. GM1‐dMOF@PTL exhibited significantly higher internalization by pro‐inflammatory M1 macrophages compared to M0 and anti‐inflammatory M2 macrophages, confirming a strong homotypic targeting effect mediated by the engineered membrane (Figure 4J,K). While the highest selectivity was observed for M1 macrophages, moderate internalization by unpolarized M0 macrophages was also noted. Biologically, this M0 uptake is expected, as unpolarized M0 precursors share baseline macrophage‐specific adhesion molecules with the M1 biomimetic coating and possess an inherently high phagocytic capacity. Importantly, from a therapeutic perspective, nanoparticle internalization by M0 cells serves as a strategic advantage rather than an off‐target effect. In the highly inflammatory microenvironments of OA and IVDD, newly recruited M0 macrophages are rapidly polarized into the pathogenic M1 phenotype. Delivering the NF‐κB inhibitor PTL to these M0 precursors proactively prevents their subsequent pro‐inflammatory polarization. Furthermore, the nanosystem demonstrated remarkable specificity for its target cells over resident IVD and cartilage cells. Uptake of GM1‐dMOF@PTL by M1 macrophages was substantially greater than that by NPC, chondrocytes (Chon), and annulus fibrosus cells (AFC) (Figure 4L,M). GM1‐dMOF@PTL was further pretreated with trypsin to digest the surface proteins. Cellular uptake analysis showed that trypsin resulted in a significant reduction in internalization by pro‐inflammatory M1 macrophages, demonstrating the crucial role of the M1 macrophage membrane proteins in targeted uptake (Figure S10A,B). The intracellular fate of the nanoparticles was tracked to assess the function of the DSPE‐PEG‐NH_2_ modification. Confocal microscopy revealed distinct trafficking patterns for the two formulations (Figure 4N). Nanoparticles lacking DSPE‐PEG‐NH_2_ (GM1‐MOF@PTL) showed sustained colocalization with LysoTracker‐labeled lysosomes, indicating GM1‐MOF@PTL were trapped within this compartment. Conversely, the DSPE‐PEG‐NH_2_‐functionalized GM1‐dMOF@PTL nanoparticles demonstrated successful lysosomal escape, as their DiD signal dissociated from the lysosomal signal over 4 h (Figure 4N). This confirms the lysosomal escape function of GM1‐dMOF@PTL.

To assess whether the biomimetic coating could overcome the challenge of rapid clearance in vivo, we tracked the retention of nanoparticles in mouse models of IVD degeneration and OA via fluorescence imaging. In the discs, intra‐discal injection of GM1‐dMOF@PTL resulted in a robust and sustained fluorescence signal at the injection site for over 6 days. In stark contrast, the signal from uncoated dMOF@PTL diminished rapidly and was barely detectable on day 6 (Figure S11A). A similar trend was observed in the knee joint, where intra‐articular injection of GM1‐dMOF@PTL led to markedly prolonged joint retention compared to the rapidly cleared dMOF@PTL (Figure S11B). Additionally, the systemic biosafety of the nanosystem was comprehensively evaluated. In vitro, GM1‐dMOF@PTL exhibited minimal cytotoxicity toward macrophages at concentrations up to 20 µg/mL (Figure S12A). In vivo toxicology studies following local injection revealed no evidence of adverse effects. H&E staining of major organs (heart, liver, spleen, lung, kidney) showed no histopathological abnormalities (Figure S12B). Blood biochemical analysis confirmed that levels of liver function markers (ALT, AST) and kidney function markers (UREA, CREA) remained within the normal physiological range (Figure S12C). Furthermore, routine blood analysis showed no significant alterations in hematological parameters, including red blood cell (RBC), white blood cell (WBC), lymphocyte, and neutrophil counts (Figure S12D). Collectively, these findings affirm the excellent biocompatibility and systemic safety of the GM1‐dMOF@PTL nanosystem.

### 3.7 GM1‐DMOF@PTL Alleviates Macrophage Pyroptosis and Restores ECM Homeostasis in Co‐Cultured NPCs and Chondrocytes via the NF‐κB/NLRP3 Inflammasome Axis

**FIGURE 5:**
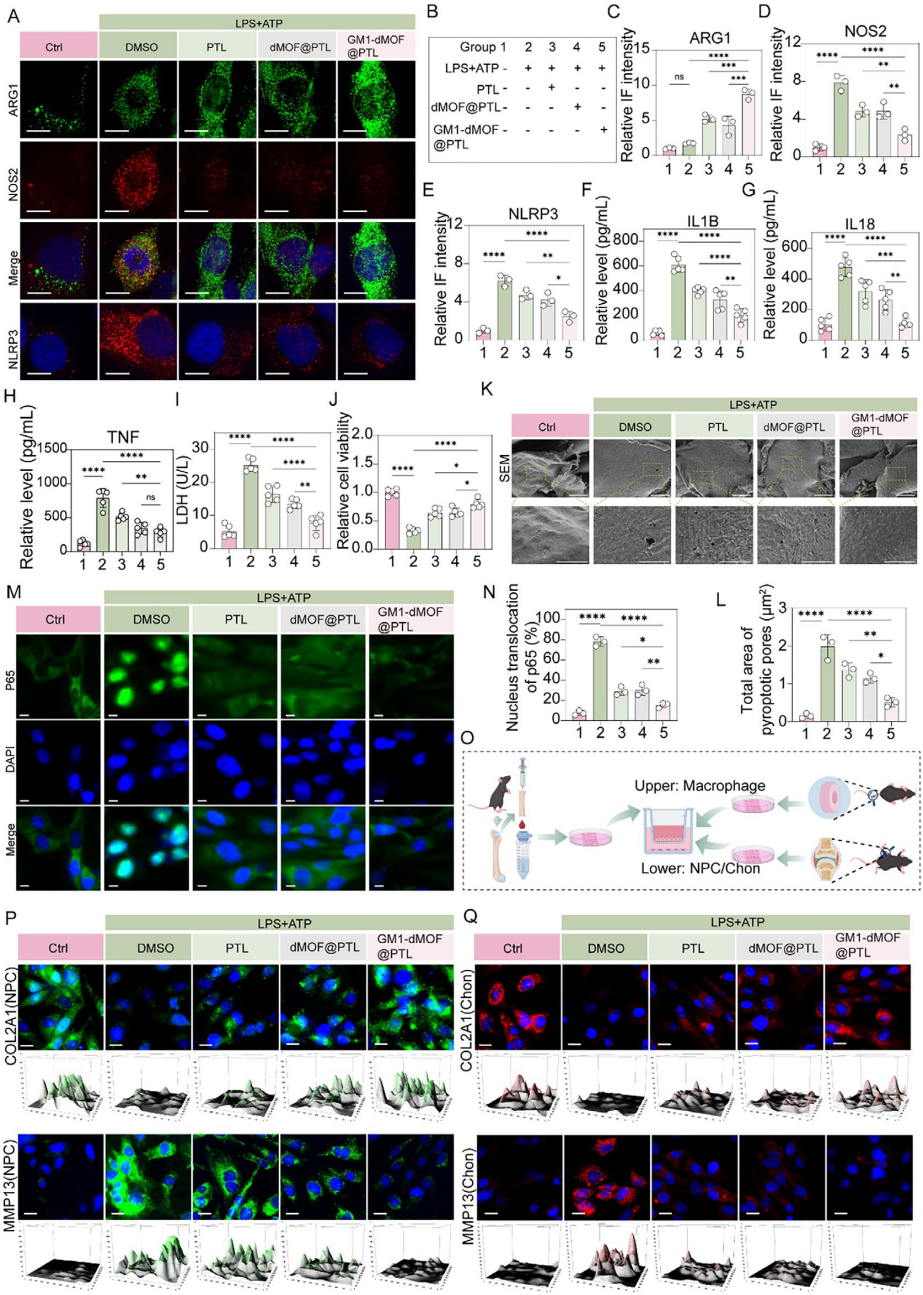
GM1‐dMOF@PTL attenuates macrophage pyroptosis by inhibiting the NF‐κB/NLRP3 inflammasome axis and reprogramming macrophage polarization to protect NPCs and chondrocytes. (A) Representative IF staining images for NOS2 (pro‐inflammatory marker), ARG1 (anti‐inflammatory marker), and NLRP3 in macrophages after various treatments (n = 3). Scale bar, 20 µm. (B) Grouping table. (C–E) Quantitative analysis of NOS2, ARG1, and NLRP3 fluorescence intensity (n = 3). (F–H) Secretion levels of IL1B, IL18, and TNF in macrophage supernatants (n = 5). (I, J) Quantitative analysis of LDH release and cell viability (n = 5). (K, L) Representative SEM images and quantification analysis showing macrophage morphology and the formation of pyroptotic pores (n = 3). Scale bar, 10 µm. (M, N) Representative IF staining images and quantitative analysis for P65 nuclear translocation (n = 3). Scale bar, 20 µm. (O) Schematic diagram of the macrophage‐NPC/chondrocyte transwell co‐culture system. (P, Q) Representative IF staining images and quantitative analysis for COL2A1 and MMP13 in co‐cultured NPCs and chondrocytes (n = 3). Scale bar, 20 µm. Data are presented as mean ± SD. The comparisons between multiple groups were conducted using one‐way ANOVA followed by Tukey's post hoc test. *, *p* < 0.05; **, *p* < 0.01; ***, *p* < 0.001; ****, *p* < 0.0001; ns = not significant.

To investigate the immunomodulatory effects of GM1‐dMOF@PTL, macrophages were stimulated with LPS+ATP to induce a pro‐inflammatory M1 phenotype. IF staining revealed that when compared with DMSO, PTL, and dMOF@PTL, GM1‐dMOF@PTL treatment significantly reversed LPS+ATP‐induced upregulation of the pro‐inflammatory marker NOS2 and downregulation of the anti‐inflammatory marker ARG1 (Figure 5A–D). Further investigation into the inflammatory cascade showed that GM1‐dMOF@PTL effectively suppressed the activation of the NLRP3 inflammasome (Figure 5A,E) and reduced the secretion of downstream pro‐inflammatory cytokines Interleukin‐1β (IL1B), Interleukin‐18 (IL18), and TNF (Figure 5F–H). Concurrently, GM1‐dMOF@PTL markedly attenuated cellular damage, as indicated by a significant decrease in LDH release and a corresponding restoration of cell viability (Figure 5I,J). SEM analysis provided crucial ultrastructural evidence linking this biochemical rescue directly to physical membrane preservation. Macrophages subjected to LPS+ATP stimulation exhibited severe morphological hallmarks of programmed lytic death, specifically the formation of numerous characteristic gasdermin‐induced membrane pores and overall membrane compromise. In stark contrast, treatment with GM1‐dMOF@PTL robustly protected the cells, maintaining a continuous, intact membrane architecture and preventing the formation of pyroptotic pores (Figure 5K,L). This ultrastructural preservation directly explains and corroborates the observed reduction in LDH and cytokine leakage. RT‐qPCR analysis revealed that GM1‐dMOF@PTL substantially reversed the LPS+ATP stimulation‐induced pro‐inflammatory M1 macrophage phenotype and concurrently promoted a shift toward an anti‐inflammatory M2 phenotype, characterized by the marked downregulation of pyroptosis‐ and inflammation‐associated genes, including *Asc, Nlrp3, Tnf, Nos2*, and *Il1b*, and elevated expression of anti‐inflammatory indicators *Il10, Mrc1*, and *Arg1* (Figure S13A).

Furthermore, to confirm the underlying mechanism, we examined the NF‐κB signaling pathway and its role in modulating pyroptosis. IF analysis demonstrated that GM1‐dMOF@PTL effectively inhibited the nuclear translocation of the p65 subunit (Figure 5M,N). Then, we further constructed a macrophage model with constitutive NF‐κB activation via p65 overexpression (P65‐OE). The overexpression efficiency of P65 was verified by Western blot (Figure S13B). IF analysis revealed that P65 overexpression induced a marked upregulation of NOS2 and ARG1, with a concurrent elevated expression of the inflammasome component NLRP3 (Figure S13C–F). The preliminary increase in ARG1 (an anti‐inflammatory gene) expression suggests the activation of an inflammatory state. This pro‐inflammatory state was substantially reversed by treatment with either the known NF‐κB inhibitor JSH‐23 or our GM1‐dMOF@PTL formulation (Figure S13C–F). Congruently, GM1‐dMOF@PTL‐induced inhibition of the NF‐κB/NLRP3 axis translated directly to a functional attenuation of pyroptotic activity, as evidenced by a dramatic reduction in the secretion of the downstream pro‐inflammatory cytokines IL1B, IL18, and TNF (Figure S13G–I). These findings confirm that GM1‐dMOF@PTL effectively mitigates macrophage inflammation and pyroptosis by directly inhibiting the NF‐κB/NLRP3 inflammasome signaling cascade.

Having established that our nanosystem profoundly suppresses TNF secretion at the upstream macrophage level, we next sought to provide direct functional evidence linking this specific molecular event to the downstream preservation of ECM in NPCs and chondrocytes via suppressing the TNF‐TNFR axis. To faithfully simulate the pathogenic paracrine microenvironment of OA and IVDD in vitro, we established a Transwell co‐culture system comprising macrophages in the upper chamber and resident structural cells (NPCs or chondrocytes) in the lower chamber (Figure S13J). As anticipated, co‐culture with pro‐inflammatory M1 macrophages induced a profound catabolic shift in both NPCs and chondrocytes, characterized by a drastic transcriptional downregulation of *Col2a1* and a concomitant pathological surge in *Mmp13* (Figure S13K–N). Crucially, targeted intervention with 5 µM R‐7050, a specific TNF receptor (TNFR) antagonist applied to NPCs or chondrocytes, substantially reversed ECM‐degrading alterations. This outcome supports a major functional contribution of macrophage‐derived TNF/TNFR signaling. Most notably, pre‐treatment of the upper‐chamber M1 macrophages with GM1‐dMOF@PTL completely recapitulated the protective efficacy of direct TNFR blockade, restoring *Col2a1* and suppressing *Mmp13* expression in both NPCs and chondrocytes to levels comparable to the R‐7050 group (Figure S13K–N).

Subsequently, to evaluate the functional consequences of macrophage pyroptosis on the ECM homeostasis of NPCs and chondrocytes, a transwell co‐culture system was employed (Figure 5O). NPC and chondrocytes co‐cultured with GM1‐dMOF@PTL‐treated macrophages exhibited a profound anabolic response, characterized by a significant upregulation in the expression of COL2A1 and a marked downregulation of MMP13 (Figure 5P,Q). RT‐qPCR analysis revealed that GM1‐dMOF@PTL restored the LPS+ATP‐induced ECM catabolic imbalance in both NPCs and chondrocytes, manifested by the increase in *Acan*, *Col2a1*, and remarkable downregulation of *Mmp13*, *Adamts5* (Figure S14A,B). These findings indicate that the GM1‐dMOF@PTL‐mediated anti‐pyroptosis effect in macrophages creates a microenvironment conducive to maintaining ECM homeostasis of NPCs and chondrocytes.

### 3.8 Attenuation of Pyroptotic Inflammation and Reversal of Nociceptive Hypersensitivity in IVDD by Homologous‐Targeting PTL Delivery

**FIGURE 6:**
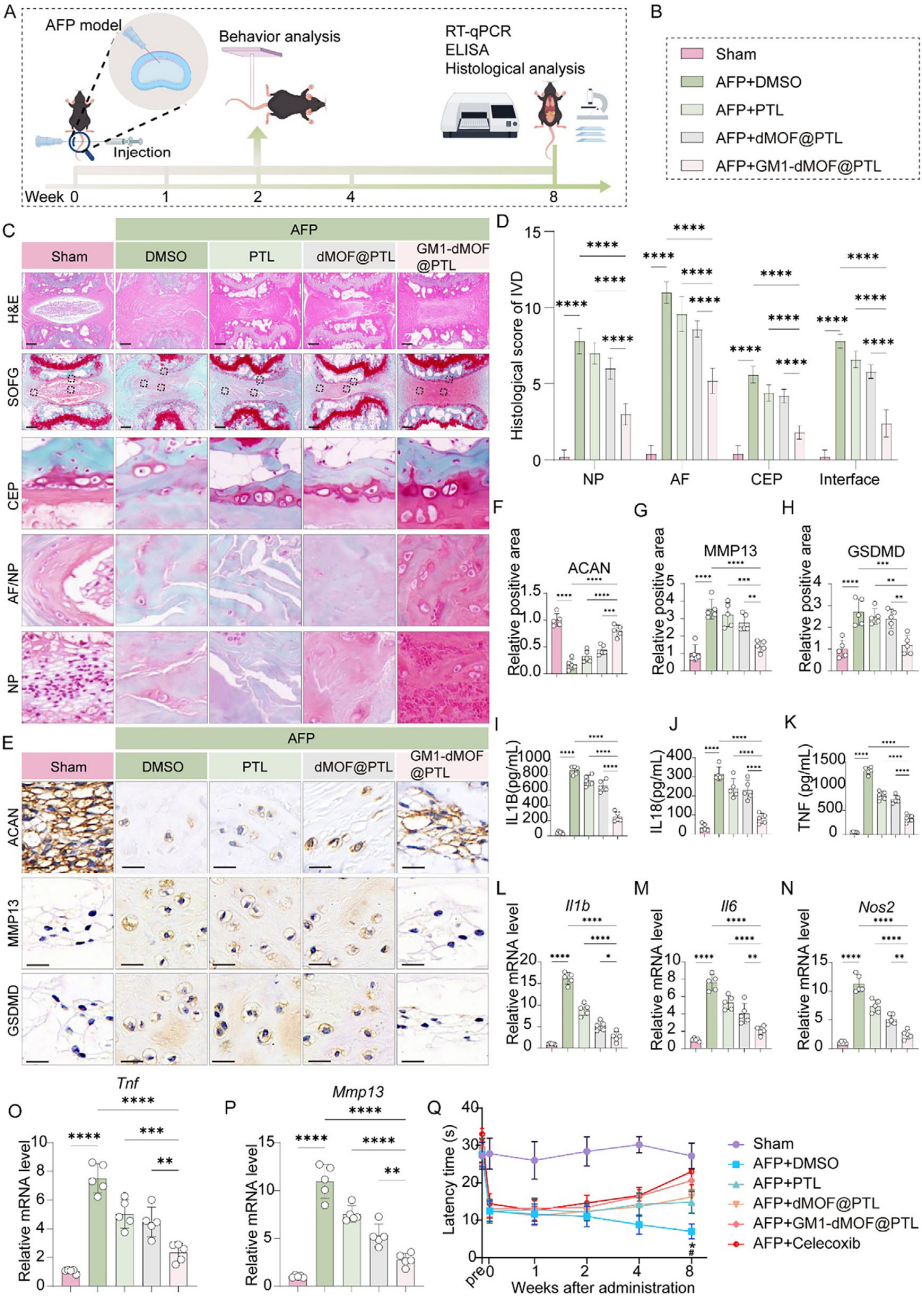
GM1‐dMOF@PTL ameliorates disc degeneration by suppressing pyroptotic inflammation in an AFP‐induced IVDD mouse model. (A) The mice experimental design diagram. (B) Schematic illustrating the experimental groups. (C) Representative H&E and SOFG staining images of intervertebral discs from each treatment group (n = 5). Scale bar, 100 µm. (D) Histological scoring of disc degeneration in NP, AF, CEP, and NP/AF interface (n = 5). (E) Representative IHC staining for the matrix protein ACAN, the catabolic enzyme MMP13, and the pyroptosis executor GSDMD in disc sections (n = 5). Scale bar, 100 µm. (F–H) Quantitative analysis of ACAN, MMP13, and GSDMD relative positive area (n = 5). (I–K) ELISA analysis of pro‐inflammatory cytokines IL‐1β, IL‐18, and TNF‐α in disc tissues from each group (n = 5). (L–P) Relative mRNA expression of key pro‐inflammatory cytokines (*Il1b, Il6, Nos2, Tnf*) and a catabolic mediator (*Mmp13*) in IVD tissues from each treatment group, as determined by RT‐qPCR (n = 5). (Q) Assessment of pain‐related behavior via measurement of thermal withdrawal latency over an 8‐week period following intra‐discal injection. The Celecoxib‐treated group served as a positive control for analgesia (n = 5). Data are presented as mean ± SD. The comparisons between multiple groups were conducted using one‐way ANOVA followed by Tukey's post hoc test. Statistical comparisons of the time‐course data were performed using a two‐way ANOVA followed by Tukey's post hoc test. *p < 0.05, **p < 0.01, ***p < 0.001, ****p < 0.0001. For subpart “Q,” *, *p* < 0.05 (represents the comparison between AFP+GM1‐dMOF@PTL vs AFP+dMOF@PTL); #, *p* < 0.05 (represents the comparison between AFP+GM1‐dMOF@PTL vs AFP+PTL).

To investigate the in vivo therapeutic efficacy of GM1‐dMOF@PTL in IVDD, we established an AFP‐induced IVDD model in mice, followed by intra‐discal injections and subsequent behavioral and histological analyses (Figure 6A). The grouping was presented (Figure 6B). We first evaluated the histological changes in the intervertebral discs eight weeks post‐surgery. H&E and SOFG staining revealed that in contrast to the intact sham discs, the AFP model exhibited severe structural degradation, characterized by a disorganized annulus fibrosus, a collapsed nucleus pulposus, and a profound loss of proteoglycans (Figure 6C). Notably, treatment with GM1‐dMOF@PTL substantially preserved the IVD architecture, maintaining a well‐defined NP/AF boundary and robust proteoglycan content, which was quantitatively confirmed by significantly lower histological scores in NP, AF, CEP, and interface compared to all other AFP‐induced groups with DMSO, PTL, or dMOF@PTL treatment (Figure 6C,D). It is imperative to note that severe IVDD is pathologically characterized not only by cellular dysfunction but also by profound extracellular matrix remodeling, specifically severe dehydration, proteoglycan depletion, and extensive tissue fibrosis. Importantly, IHC staining accurately reflected these profound microenvironmental shifts (Figure 6E). In the highly degenerated IVDD models (DMSO, PTL, and dMOF@PTL groups), the extracellular matrix transformed into a dense fibrotic texture, accompanied by severe hypocellularity. Within these stressed, fibrotic regions, the remaining clustered cells exhibited a dramatic loss of ACAN and pronounced upregulation of the catabolic enzyme MMP13 and the pyroptosis executor GSDMD. Remarkably, these pathological shifts were profoundly ameliorated by GM1‐dMOF@PTL treatment. GM1‐dMOF@PTL not only suppressed the expression of these catabolic and pyroptotic markers in the resident cells, but also effectively restored local cellularity and prevented the pathological fibrotic transition of the matrix, maintaining a highly hydrated histological profile closely resembling that of healthy discs (Figure 6E–H). Consistent with these histological findings, ELISA analysis demonstrated that the elevated levels of pro‐inflammatory cytokines IL1B, IL18, and TNF in the discs of the AFP‐induced IVDD were more remarkably suppressed by the GM1‐dMOF@PTL treatment (Figure 6I–K). The impact of our nanosystem on the inflammatory and catabolic microenvironment within the IVDs was also assessed using RT‐qPCR. The AFP model successfully induced a robust degenerative phenotype, characterized by a significant upregulation in the relative mRNA expression of key pro‐inflammatory cytokines, including *Il1b*, *Il6*, *Tnf*, and *Nos2*, and catabolic mediator *Mmp13* (Figure 6L–P). The expression levels of *Il1b*, *Il6*, *Nos2*, *Tnf*, and *Mmp13* in the GM1‐dMOF@PTL‐treated group were significantly lower than those in the PTL and dMOF@PTL groups and were restored to levels approaching those of the healthy Sham group (Figure 6L–P). These findings indicate that the GM1‐dMOF@PTL nanosystem effectively mitigates the inflammatory and catabolic cascade at the transcriptional level.

Crucially, we sought to determine whether this potent molecular modulation translated into functional improvement, specifically the alleviation of pain‐related behavior. We assessed hyperalgesia by measuring withdrawal latency time over an 8‐week period following treatment administration (Figure 6Q). As a positive control for analgesia, a group was treated with Celecoxib, a standard non‐steroidal anti‐inflammatory drug (NSAID). Prior to treatment, all AFP‐injured mice exhibited significantly reduced latency times compared to the Sham group, confirming the successful establishment of hyperalgesia. Over the 8‐week study, the vehicle‐treated (AFP+DMSO) group showed a progressive decline in latency time, indicating worsening pain. In contrast, the positive control group treated with Celecoxib demonstrated a sustained reversal of hyperalgesia. While the PTL and dMOF@PTL treatments offered modest and transient pain relief, the GM1‐dMOF@PTL treatment resulted in a remarkable and durable recovery. From the first week post‐injection, mice treated with GM1‐dMOF@PTL showed a progressive increase in withdrawal latency (Figure 6Q). Collectively, these data demonstrate that the administration of GM1‐dMOF@PTL not only ameliorates the degenerative molecular milieu but also provides robust and long‐lasting relief from discogenic pain.

### 3.9 Preservation of Joint Integrity and Alleviation of Degenerative Pain by Macrophage‐Biomimetic GM1‐dMOF@PTL

**FIGURE 7:**
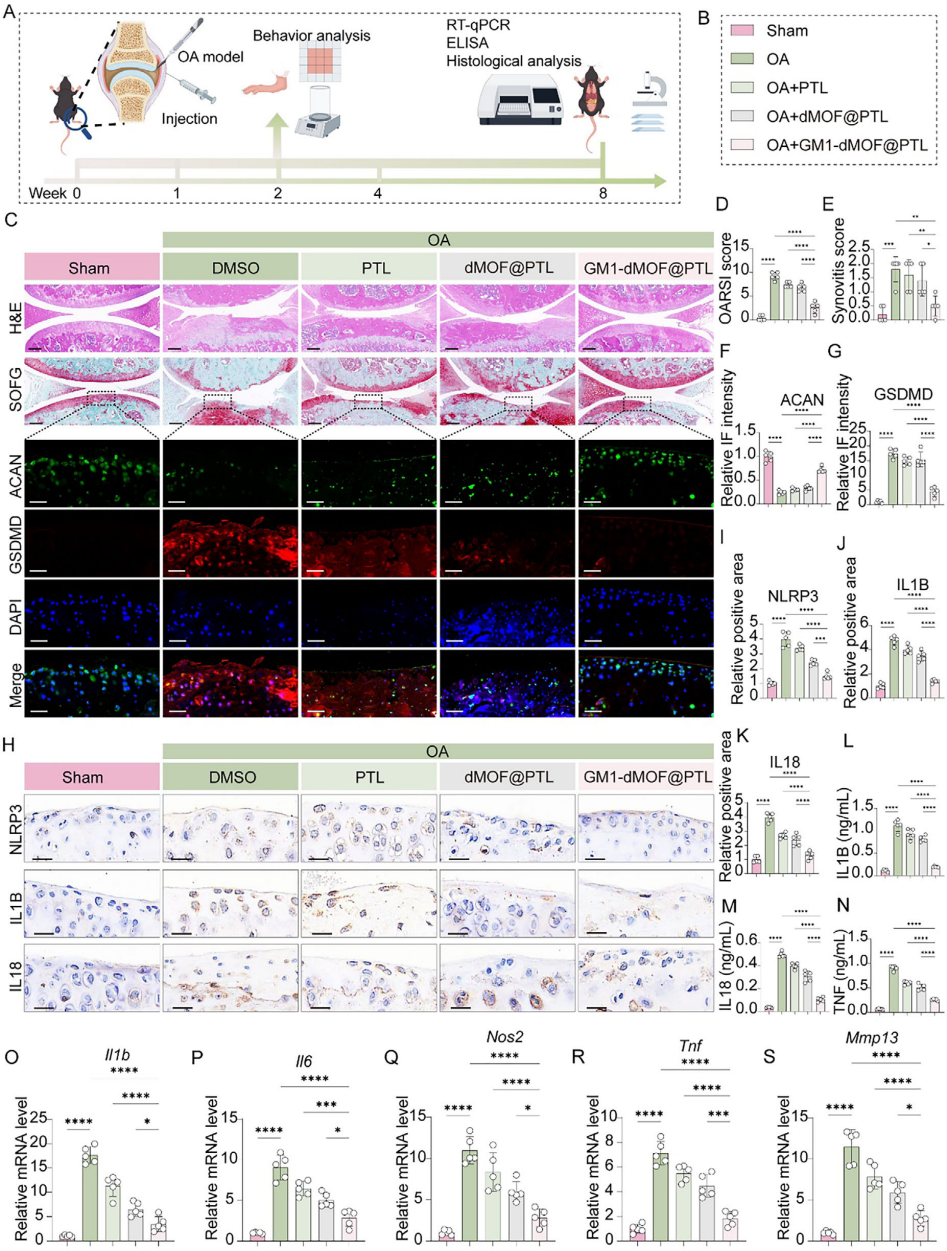
GM1‐dMOF@PTL attenuates OA progression by suppressing pyroptotic inflammation and preserving joint integrity. (A) The mice experimental design diagram. (B) Schematic illustrating the experimental groups. (C) Representative histological images of knee joint sections stained with H&E, SOFG, and representative IF images of ACAN and GSDMD at 8 weeks post‐surgery (n = 5). Scale bars, 100 µm. (D, E) Quantitative OARSI score and synovitis score evaluating the severity of joint degeneration (n = 5). (F, G) Quantitative analysis for IF intensity of ACAN and GSDMD (n = 5). (H–K) Representative IHC staining images and corresponding quantitative analysis of NLRP3, IL1B, and IL18 in knee joint sections (n = 5). Scale bars, 40 µm. (L–N) Levels of pro‐inflammatory cytokines IL1B, IL18, and TNF in articular cartilage tissue lysates (n = 5). (O–S) Relative mRNA expression of key pro‐inflammatory cytokines (*Il1b, Il6, Nos2, Tnf*) and a catabolic mediator (*Mmp13*) in knee joint tissues from each treatment group (n = 5). Data are presented as mean ± SD. Statistical comparisons were performed using one‐way ANOVA followed by Tukey's post hoc test. *p < 0.05, **p < 0.01, ***p < 0.001, ****p < 0.0001.

To investigate the therapeutic efficacy of GM1‐dMOF@PTL in OA, we established a surgically induced OA model in mice, followed by a regimen of intra‐articular injections, behavioral analyses, and terminal histological evaluation (Figure 7A). The grouping was presented (Figure 7B). The gross histological changes in the knee joints eight weeks post‐surgery were evaluated. H&E and SOFG staining showed that in the sham group, the articular cartilage remained smooth and intact with robust proteoglycan content. Conversely, the OA model group exhibited severe joint degradation, characterized by extensive cartilage erosion, fibrillation, and a profound loss of proteoglycans (Figure 7C). Notably, treatment with GM1‐dMOF@PTL substantially attenuated these degenerative changes, preserving a smoother cartilage surface and a significantly greater proteoglycan‐rich matrix (Figure 7C). This protective effect was quantitatively validated by significantly lower OARSI score and synovitis score in the GM1‐dMOF@PTL group compared to all other OA‐induced groups (Figure 7D,E). Additionally, we assessed the molecular and cellular changes within the joint tissue. IF staining revealed that the marked loss of the anabolic marker ACAN and the strong upregulation of GSDMD in OA chondrocytes were both remarkably ameliorated by GM1‐dMOF@PTL treatment (Figure 7F,G). IHC analysis showed that the positive areas of NLRP3, IL1B, and IL18 were increased in the OA group, while GM1‐dMOF@PTL remarkably lowered the levels of NLRP3, IL1B, and IL18 when compared with the PTL‐ or dMOF@PTL‐treated mice (Figure 7H–K). ELISA analysis of articular cartilage samples in mice demonstrated that the elevated levels of pro‐inflammatory cytokines IL1B, IL18, and TNF in the OA group were significantly suppressed following GM1‐dMOF@PTL administration (Figure 7L–N). As expected, RT‐qPCR analysis revealed that the mRNA levels of *Il1b, Il6, Nos2, Tnf*, and *Mmp13* in the GM1‐dMOF@PTL‐treated OA mice were significantly lower than those in the PTL and dMOF@PTL groups (Figure 7O–S).

To substantiate our histological and molecular findings with definitive functional outcomes, we employed a comprehensive suite of validated behavioral assays to evaluate nociceptive sensitization and mobility. The therapeutic efficacy in alleviating nociceptive sensitization of our formulation was assessed. The induction of OA led to significant locomotor impairments, characterized by statistically significant reductions in active time, distance travelled, central mean speed, and mean speed in an OFT analysis compared to the sham group. Treatment with the GM1‐dMOF@PTL formulation effectively reversed locomotor deficits (Figure S15A–E). Additionally, mice in the OA group also developed pronounced pain‐related behaviors, evident as progressive thermal hyperalgesia (decreased Paw Withdrawal Latency, PWL) and mechanical allodynia (decreased Paw Withdrawal Threshold, PWT) over an 8‐week period (Figure S15F,G). The administration of GM1‐dMOF@PTL provided sustained and potent analgesia, markedly attenuating the development of both thermal and mechanical hypersensitivity throughout the study (Figure S15F,G). The therapeutic efficacy of GM1‐dMOF@PTL in restoring joint function was superior to that of free PTL or non‐targeted dMOF@PTL and was comparable to the positive control, celecoxib, highlighting its potential for treating OA‐associated pain and mobility dysfunction.

## 4 Discussion

In the present study, we have unmasked a conserved, pro‐inflammatory M1 macrophage subpopulation as a central pathological driver common to both human OA and IVDD tissues, a critical insight gained from our scRNA‐seq analysis. To address this common enemy, we engineered a novel macrophage‐biomimetic nanosystem, GM1‐dMOF@PTL, designed not merely for drug delivery, but for a precision‐guided mission to neutralize these pathogenic cells. Our findings validate this nanosystem's role as a master regulator of the inflammatory microenvironment, functioning as an advanced immunoregulatory platform executing a sophisticated multi‐pronged therapeutic intervention.

Mechanistically, the success of GM1‐dMOF@PTL lies in its meticulously orchestrated, three‐tiered therapeutic strategy. First, leveraging its biomimetic camouflage, the membrane facilitates highly specific homotypic targeting and ensures prolonged retention within the complex pathological microenvironment of the diseased joint or disc. Second, serving as an extracellular interceptor, the engineered IL‐1R2 decoy receptor effectively attenuates paracrine signaling by neutralizing the pivotal pro‐inflammatory cytokine, IL‐1β. Finally, upon successful infiltration, the encapsulated drug, PTL, is delivered intracellularly after lysosomal escape to potently inhibit the NF‐κB/NLRP3 inflammasome axis, thereby dismantling the cell's pyroptotic cascade. More importantly, this intervention achieves the ultimate therapeutic goal: it “reprograms” the pathogenic macrophage from a pro‐inflammatory driver into a reparative mediator. The local administration of GM1‐dMOF@PTL protected mice against both OA and IVDD progression, restoring ECM homeostasis and providing sustained pain relief, thus demonstrating the successful translation of this targeted cellular reprogramming strategy from concept to compelling in vivo efficacy.

To the best of our knowledge, the present study is the first to leverage single‐cell transcriptomics to identify a shared cellular target between OA and IVDD and subsequently design a unified nanotherapeutic strategy to address it. Previous scRNA‐seq studies have indeed identified pro‐inflammatory macrophage subsets as key pathogenic players in both OA and IVDD independently [30, 31]. However, our study is the first to perform an integrated analysis, revealing a conserved transcriptomic signature that explicitly links the two pathologies through a common cellular driver. This finding moves beyond treating OA and IVDD as disparate conditions and provides a molecular rationale for a unified therapeutic approach. While both diseases are known to be inflammation‐driven, therapies have historically targeted broad inflammatory pathways, such as NSAIDs, or individual cytokines, such as anti‐TNF‐α biologics, often with limited efficacy and significant off‐target effects [32]. Our approach represents a paradigm shift from nonspecific anti‐inflammatory treatment to precision cellular reprogramming. This contrasts with other targeted strategies that rely on conjugating antibodies to specific surface markers like CD44 or folate receptors [33, 34], which can have variable expression levels. Homotypic targeting, by leveraging the full suite of membrane adhesion molecules, may offer a more robust and biologically relevant approach to engaging pathogenic cells. By identifying and targeting the specific pathogenic pro‐inflammatory macrophage signature, we aim not just to dampen inflammation, but to promote the pro‐inflammatory to anti‐inflammatory transition, a strategy that holds greater promise for restoring tissue balance. The ability of GM1‐dMOF@PTL to preferentially internalize into pro‐inflammatory macrophages underscores the precision of this approach.

Classically, nanomedicine design focuses on overcoming pharmacokinetic hurdles, and the use of cell membrane‐camouflaged nanoparticles is a well‐established strategy to improve biocompatibility and circulation time [35, 36]. Our work advances this concept by creating a bioactive camouflage that participates directly in therapy. While some studies have explored genetically engineering source cells to display specific ligands or antigens [37], the integration of a decoy receptor like IL‐1R2 onto a macrophage membrane for synergistic therapy is a significant innovation. This dual‐action approach, sequestering extracellular IL‐1β while simultaneously delivering an intracellular inhibitor, is a key differentiator from therapies that only do one or the other, such as systemic IL‐1β antibody or nanoparticles simply delivering an anti‐inflammatory drug [38]. The design transforms the nanoparticle into a molecular sponge, a concept previously explored using liposomes [39], but our system uniquely combines this extracellular neutralization with the intrinsic homing properties of a pathogenic macrophage membrane. Furthermore, the PTL‐loaded MOF core was deliberately modified with DSPE‐PEG‐NH_2_ to facilitate lysosomal escape via the “proton sponge” effect [40], a well‐validated mechanism to ensure the cytosolic delivery of payloads. Our findings revealed that this strategy ensures that PTL can effectively inhibit the NF‐κB pathway, rather than being degraded. The combination of these three elements, homotypic targeting, decoy receptor action, and payload delivery, creates a multi‐pronged attack on the inflammatory cascade that is more sophisticated than previously reported single‐modality nanotherapeutics.

The rapid clearance of therapeutics from the joint and disc is a major clinical barrier [41, 42]. While other nanoparticle platforms such as pH‐responsive sustained‐release systems and matrix metalloproteinase‐responsive nanoparticles have shown improved retention over free drugs [43, 44], our observed retention of over 6 days is highly favorable and compares well with other MOF systems, which were designed for promoting diabetic wound healing and showed a cumulative release rate of 81.4% in 30 min [45]. This sustained local concentration is critical for interrupting chronic disease processes. Our finding aligns with the overarching goal of advanced delivery systems, such as those based on MOF, which aim to prolong intra‐articular residency. For instance, a recently developed MOF‐based nanogel (KZIF@HA) achieved sustained drug release for over one month by forming a hydrophilic network that minimized leakage from the joint cavity [46]. While that system represents an exceptional example of long‐term retention, it is noteworthy that its mechanism relies on the formation of a nanogel. This strategy is highly effective within the spacious synovial cavity but is largely incompatible with the micro‐architectural constraints of the intervertebral disc, which might lack a significant injectable void for gel formation [47]. In this challenging context, our platform's ability to remain localized for over six days as a nanoparticle dispersion is not only strategically sufficient but also more appropriate for the target tissue. This was demonstrated by the successful therapeutic outcome achieved with a practical weekly injection regimen (two injections total), validating our design approach. This sustained local concentration is critical for interrupting chronic disease processes. The encapsulation of PTL, a potent but poorly soluble NF‐κB inhibitor, aligns with a broader strategy in nanomedicine to repurpose promising natural compounds. Previous studies have encapsulated PTL to treat conditions like cancer or systemic inflammation [48, 49], but its targeted delivery specifically to pathogenic macrophages in degenerative joint and spine disease represents a novel and highly relevant application. By encapsulating PTL and restricting its delivery to the cells driving the pathology, we maximize its therapeutic index while minimizing risk, elegantly solving its “delivery problem.”

Our research demonstrated that the GM1‐dMOF@PTL nanosystem can dramatically inhibit the pyroptotic signaling cascade in macrophages. This is particularly salient, as the NLRP3 inflammasome‐GSDMD pathway has been increasingly implicated as a central driver of the sterile inflammation that characterizes both OA and IVDD [50, 51]. Other nanotherapeutics have been designed to inhibit the NLRP3 inflammasome [51, 52], but our system is unique in that it couples this inhibition with precise cellular targeting and extracellular cytokine neutralization. The therapeutic efficacy was critically dependent on the biomimetic coating, as evidenced by trypsin digestion experiments that abolished targeted uptake. This finding is consistent with other studies on homotypic targeting systems, confirming that the therapeutic benefit is not merely due to the drug payload but is fundamentally driven by the engineered biological interface [53]. Additionally, the moderate uptake by unpolarized M0 macrophages provides a preemptive therapeutic advantage by blocking their impending polarization into the pro‐inflammatory M1 phenotype within the diseased microenvironment. While early nanotherapeutic internalization by M0 macrophages raises theoretical considerations regarding the potential impairment of their normal physiological phagocytic functions required for tissue repair, this risk is mitigated by the highly specific pharmacological mechanism of PTL. PTL specifically silences NF‐κB‐driven inflammatory cascades and inflammasome activation without disrupting the cytoskeletal actin dynamics essential for basic cellular engulfment. Furthermore, by preemptively skewing these macrophages away from a catabolic M1 state and toward a pro‐resolving M2‐like phenotype, the intervention likely preserves, or even enhances, their capacity for efferocytosis—the beneficial clearance of apoptotic debris—thereby facilitating matrix repair without compromising fundamental innate immunity. Thus, we clarified that the therapeutic mechanism of our nanosystem in OA and IVDD was due to the selective reprogramming of pathogenic macrophages, achieved through the synergistic action of homotypic targeting, decoy receptor neutralization, and intracellular NF‐κB inhibition.

Furthermore, it is important to contextualize the 8‐week in vivo evaluation period utilized in our study to define therapeutic success. While OA and IVDD are chronic conditions that progress over years or decades in humans, murine surgical models, specifically the surgical instability OA model and the AFP IVDD model, induce a vastly accelerated pathological cascade. In these models, 8 weeks post‐surgery is universally recognized as a standard endpoint representing advanced, severe structural degeneration. Recent authoritative studies have consistently demonstrated that by 8 weeks, untreated mice exhibit end‐stage hallmarks, including near‐complete cartilage erosion, severe disc space collapse, and extensive tissue fibrosis replacing native resident cells [54, 55, 56, 57]. Clinically, a treatment is classified as “disease‐modifying” rather than merely symptomatic if it fundamentally a this structural deterioration and preserves ECM integrity. Palliative treatments may temporarily mask pain, but they fail to prevent joint or disc destruction, whereas true disease modification requires the prevention of the structural lesions that ultimately drive nociceptive behavior [57]. In our study, the robust structural preservation observed at the 8‐week end‐stage time point demonstrates that GM1‐dMOF@PTL intervenes at the fundamental pathophysiological level. Notably, even in clinical settings, 8 weeks serves as a critical milestone for assessing the primary therapeutic response in OA patients [58]. Consequently, our comprehensive structural and functional data support that the GM1‐dMOF@PTL nanosystem provides disease‐modifying potential, effectively arresting the degenerative cascade rather than merely offering a transient delay of symptoms.

Despite the promising therapeutic outcomes presented, our study has several limitations that should be acknowledged. Our investigations were primarily conducted in rodent models of surgically induced OA and IVDD. While these models are well‐established for studying disease mechanisms and therapeutic responses, they may not fully recapitulate the complex biomechanics and chronic, low‐grade inflammatory milieu characteristic of slowly progressing, age‐related degenerative disease in humans. Furthermore, while our study provides compelling evidence for the central role of the pathogenic macrophage and the NF‐κB/NLRP3 axis, it is important to acknowledge that other signaling networks and cell types undoubtedly contribute to the pathophysiology of these multifactorial disorders and warrant further investigation. From a translational perspective, several aspects require deeper exploration before the GM1‐dMOF@PTL nanosystem can be considered for clinical use. The long‐term biosafety, potential immunogenicity upon repeated administration, and the precise in vivo pharmacokinetic and degradation profiles of the nanosystem within the joint or disc microenvironment must be rigorously evaluated. Finally, the therapeutic efficacy in our study was evaluated up to an 8‐week endpoint. For chronic degenerative conditions like OA and IVDD, extending this observation period to 12 or 16 weeks would be crucial to assess the durability of the therapeutic effect. A longer‐term study would allow us to distinguish between a true disease‐modifying reversal of pathology versus a transient delay in progression, thereby substantially strengthening the clinical relevance of our findings. Future work should therefore prioritize validating these results in large animal models, which more closely mimic human physiology, and further refining the nanocarrier design for sustained, long‐term immunomodulation.

## 5 Conclusion

In conclusion, this study elucidates a shared macrophage‐driven pathological mechanism underlying both OA and IVDD and presents a targeted, disease‐modifying nanotherapeutic strategy. Our major finding identifies a conserved, pathogenic IL‐1β^+^ macrophage subpopulation fueled by the NF‐κB/pyroptosis axis as a central driver of extracellular matrix destruction in both diseases. Mechanistically, the engineered GM1‐dMOF@PTL nanosystem executes a highly synergistic, three‐tiered cellular reprogramming strategy: (1) homotypic targeting to precise inflammatory niches via biomimetic macrophage membranes, (2) extracellular neutralization of paracrine IL‐1β signaling via the overexpressed IL1R2 decoy receptor, and (3) intracellular dismantling of the NF‐κB/NLRP3 inflammasome cascade via the lysosome‐escaping PTL payload. From a translational perspective, local administration of this multi‐modal nanosystem attenuates structural deterioration, restores matrix homeostasis, and provides robust, sustained pain relief in murine models. This represents a critical paradigm shift from conventional, broad‐spectrum symptomatic relief to precision‐guided cellular reprogramming. Despite these promising outcomes, this study has limitations. The in vivo evaluations were conducted using surgically induced, acute rodent models over an 8‐week period, which may not fully recapitulate the biomechanical complexity and chronic, age‐related progression of these diseases in humans. Future perspectives must prioritize extending observation periods to assess long‐term therapeutic durability and potential immunogenicity, alongside rigorous pharmacokinetic and biosafety evaluations in large animal models that more closely mimic human joint and spinal physiology. The current in vivo efficacy evaluations were exclusively conducted using male C57BL/6J mice to minimize the potentially confounding variables associated with estrous cycle fluctuations. Given the well‐documented sexual dimorphism in the human epidemiology, inflammatory manifestations, and pain perception associated with both OA and IVDD, this approach limits the broader translational extrapolation of our findings. Future preclinical studies are fundamentally warranted to rigorously validate the pharmacokinetics, safety profile, and therapeutic efficacy of this biomimetic nanotherapeutic in age‐matched female animal models. Ultimately, this targeted immunomodulatory platform offers a compelling and translatable framework for developing true disease‐modifying therapies for intractable degenerative musculoskeletal disorders.

## Author Contributions

**Jiangang Shi**, **Kaiqiang Sun**, and **Fudong Li** designed the study, supervised experiments, and revised the manuscript. Fudong Li wrote the manuscript. **Tianyi Zhao**, Fudong Li, **Bin Zhang**, **Bing Zheng**, and **Zhiqiu Zhang** conducted experiments and data analysis. **Jialin Li**, **Chen Yan**, and **Linhui Han** revised and reviewed the study. Kaiqiang Sun and Tianyi Zhao provided final approval of the version to be submitted.

## Conflicts of Interest

The authors declare no conflicts of interest.

## Supporting information




**Supporting File**: advs77171‐sup‐0001‐SuppMat.docx.

## Data Availability

Raw data are available on reasonable request from the corresponding author. The data that support the findings of this study are openly available to ensure full transparency and reproducibility. The newly generated transcriptomic sequencing data have been deposited in the Genome Sequence Archive (GSA) at the National Genomics Data Center, China National Center for Bioinformation (CNCB) (https://ngdc.cncb.ac.cn/gsa), and are publicly accessible under the accession number CRA046573. The publicly available single‐cell RNA sequencing (scRNA‐seq) datasets analyzed in this study were obtained from the Gene Expression Omnibus (GEO) repository (http://www.ncbi.nlm.nih.gov/geo) under the accession numbers GSE220243 (for osteoarthritis) and GSE165722 (for intervertebral disc degeneration). All other raw data, processed data, and analytical models supporting the findings of this study are available within the article and its Supplementary Information files, or from the corresponding authors upon reasonable request.
