# Supplementary material for: A Decoy‐Receptor‐Armed Biomimetic Nanotherapeutic With Inherent Tropism for Conserved Pathogenic Macrophages for Treating Osteoarthritis and Intervertebral Disc Degeneration
Source: Adv Sci (Weinh). 2026 Aug 20:e77171. Online ahead of print. doi: 10.1002/advs.77171 (PMC13491363; doi:10.1002/advs.77171)
Supplement: Supplementary file 1 [file ADVS-9999-e77171-s001.docx]

**Figure S1**. Volcano plots of DEGs among various cell types in IVDD and OA. (A) Volcano plots of DEGs across major cell types in the IVDD dataset. (B) Volcano plots of DEGs across major cell types in the OA dataset.

**Figure S2**. Heterogeneity and pathological trajectory of NPCs subpopulations in IVDD. (A) UMAP plot illustrating eight distinct subpopulations (NPC0–NPC7) of NPCs identified through subclustering. (B) Bar plot showing the changes in the relative proportions of NPC subpopulations between mild and severe degeneration samples. (C) Volcano plots displaying DEGs for representative NPC subclusters, identifying homeostatic (NPC0), catabolic (NPC4), and fibrotic (NPC3) phenotypes. (D) Pseudotime trajectory analysis of NPC subpopulations, mapping a cellular progression from a healthy to a degenerative state. (E) Heatmap visualizing the dynamic expression of key genes along the pseudotime trajectory, revealing distinct modules associated with early-stage homeostasis and late-stage catabolism/fibrosis.

**Figure S3**. Characterization of chondrocyte subpopulation heterogeneity and degenerative progression in OA. (A) UMAP visualization of eight distinct chondrocyte subpopulations (Chon0–Chon7) identified in the OA dataset. (B) Bar plot quantifying the shifts in the proportional distribution of chondrocyte subpopulations between normal and OA tissues. (C) Volcano plots of DEGs for key chondrocyte subclusters, highlighting unique transcriptomic profiles associated with fibrotic (Chon3) and catabolic (Chon5) states. (D-E) Pseudotime trajectory analysis of chondrocyte subpopulations, delineating a pathological progression towards a degenerative endpoint. (F) Heatmap displaying gene expression dynamics along the chondrocyte pseudotime trajectory, showing progressive downregulation of matrix genes (COL2A1, ACAN) and upregulation of hypertrophic and inflammatory markers (INHBA, S100A family).

**Figure S4**. Conserved transcriptomic signatures and regulatory networks drive pathological cell states in IVDD and OA. (A) Venn diagram illustrating the overlap of 107 DEGs between the Chon5 and NPC4 subclusters. (B) KEGG pathway enrichment analysis of the shared DEGs, showing significant enrichment in inflammation-related pathways such as "TNF signaling" and "NF-kappa B signaling". (C) Transcription factor regulon analysis identifying NFIL3, FOSB, and REL as key active regulators in both Chon5 and NPC4 subclusters. (D) Venn diagram showing 89 common DEGs between the Chon3 and NPC3 subclusters. (E) KEGG pathway enrichment analysis for the shared genes, showing enrichment for "Focal adhesion" and "ECM-receptor interaction". (F) Transcription factor regulon analysis identifying TWIST1 and SOX9 as highly active in both Chon3 and NPC3 subclusters.

**Figure S5**. Subclustering reveals macrophage heterogeneity in the IVDD and OA microenvironments. (A) UMAP plot showing five distinct macrophage subpopulations (Mac0–4) identified in the IVDD dataset. (B) Feature plots illustrating the expression of key inflammatory markers such as IL1B, NLRP3 and CCL20 in macrophage subclusters. (C) UMAP plot resolving seven distinct macrophage subpopulations (Mac0–6) in the OA dataset. (D) Feature plots showing elevated expression of pro-inflammatory genes within the OA Mac0 subcluster.

**Figure S6**. Shared pro-inflammatory signaling pathways are enriched in IVDD and OA macrophages. (A) KEGG pathway enrichment analysis of the pro-inflammatory Mac0 subcluster from the IVDD dataset, demonstrating significant enrichment for the “NF-kappa B signaling pathway,” “NOD-like receptor signaling pathway,” and “TNF signaling pathway”. (B) KEGG pathway enrichment analysis of the pro-inflammatory Mac0 subcluster from the OA dataset, revealing a conserved activation of “NF-kappa B signaling pathway,” “NOD-like receptor signaling pathway,” and “TNF signaling pathway” as observed in IVDD.

**Figure S7**. Conservation of the TNF-TNFRSF1A signaling axis in pro-inflammatory macrophages of IVDD and OA. Ligand-receptor interaction analysis of the TNF signaling pathway in pro-inflammatory macrophage from both IVDD and OA. The analysis highlights the TNF-TNFRSF1A signaling pair as the predominant contributor to pathway activity.

**Figure S8**. Transcriptomic profiling reveals PTL's potent anti-inflammatory effects in macrophages. (A) Hierarchical clustering heatmap of DEGs illustrating distinct transcriptomic profiles. (B-C) GO enrichment analysis of DEGs, showing significantly enriched terms related to inflammatory pathways. Data are derived from transcriptomic analysis (n = 3).

**Figure S9**. Validation of the structural integrity, IL1R2 decoy receptor overexpression, and enhanced anti-inflammatory efficacy of the nanosystem. (A) Representative TEM images illustrating the exceptional structural stability and intact morphology of GM1-dMOF@PTL after 3 and 7 days of incubation in physiological conditions (pH 7.4 + PBS) versus a simulated acidic and inflammatory microenvironment (pH 6.5 + 10 ng/mL IL-1β). Scale bars = 50 nm. (B) Cumulative in vitro release profiles of PTL from GM1-dMOF@PTL under the corresponding physiological and acidic inflammatory conditions over 7 days (n = 3). (C-D) RT-qPCR (C) and Western blot analysis with quantification (D) confirming the successful overexpression of the IL1R2 decoy receptor in source macrophages following lentiviral transfection (vec vs. LV-*Il1r2*) (n = 3). The full-length, uncropped western blot images corresponding to the immunoblots presented in this figure are available in the Supplementary Information of western blot images. (E) Quantitative densitometric analysis of specific membrane proteins (IL1R2, CD68, and Integrin β1), confirming the successful retention of native macrophage markers and the engineered overexpression of IL1R2 on the biomimetic GM1-dMOF@PTL surface compared to native M1 macrophages and uncoated dMOF@PTL (n = 3). (F–J) Relative mRNA expression levels of key pro-inflammatory cytokines, including *Il1b, Il6, Ifng, Tnf*, and *Il18*, in IL-1β-stimulated macrophages, demonstrating the superior anti-inflammatory effect of GM1-dMOF@PTL compared to uncoated dMOF@PTL (n = 3). Data are presented as mean ± SD. The comparisons among groups were conducted using one-way ANOVA followed by Tukey’s post hoc test. The comparisons between two groups were conducted using an unpaired, two-tailed Student’s t-test. **P* < 0.05, ***P* < 0.01, ****P* < 0.001, *****P* < 0.0001.

**Figure S10**. M1 macrophage membrane proteins are crucial for the homologous targeting of GM1-dMOF@PTL. (A) Representative fluorescence images demonstrating the cellular uptake of GM1-dMOF@PTL by M1 macrophages with or without trypsin pretreatment to digest surface proteins (n = 3). Scale bar, 20 μm. (B) Quantitative analysis of fluorescence intensity, showing a significant reduction in nanoparticle internalization after trypsin digestion (n = 3). Data are presented as mean ± SD. The comparisons between two groups were conducted using a Student’s t-test. **, *P* < 0.01.

**Figure S11**. Biomimetic coating enhances in vivo retention of GM1-dMOF@PTL in mouse models of IVDD and OA. (A) Representative in vivo fluorescence images tracking the retention of Cy5.5-labeled nanoparticles following intra-discal injection in a mouse model of IVDD over 6 days (n = 5). (B) Representative in vivo fluorescence images showing prolonged joint retention of Cy5.5-labeled nanoparticles after intra-articular injection in a mouse model of OA over 6 days (n = 5).

**Figure S12**. In vitro and in vivo biosafety evaluation of the GM1-dMOF@PTL nanosystem. (A) Cell viability of macrophages treated with a gradient of GM1-dMOF@PTL concentrations for 24 hours, as assessed by CCK8 assay (n = 5). (B) Representative H&E staining images of major organs (heart, liver, spleen, lung, and kidney) from mice 8 weeks after local injection, showing no apparent histopathological damage (n = 5). Scale bar, 100 μm. (C) Serum biochemical analysis of liver function markers (ALT, AST) and kidney function markers (UREA, CREA) in mice from different treatment groups (n = 5). (D) Routine blood analysis of key hematological parameters, including white blood cell (WBC), red blood cell (RBC), lymphocyte, and neutrophil counts (n = 5). Data are presented as mean ± SD. The comparisons between groups were conducted using one-way ANOVA followed by Tukey’s post hoc test. ***, *P* < 0.001.

**Figure S13**. GM1-dMOF@PTL mitigates macrophage pyroptosis via NF-κB signaling inhibition and prevents structural cell degeneration by severing the pathogenic TNF paracrine axis. (A) Relative mRNA expression of pyroptosis-associated (*Asc* and *Nlrp3*), M1-associated (*Tnf, Nos2, Il1b*), and M2-associated (*Il10, Mrc1, Arg1*) markers (n = 3) in macrophages. (B) Western blot analysis for the overexpression efficiency of P65. (C) Representative IF staining images for ARG1, NOS2, and NLRP3 in P65-overexpressing (P65-OE) macrophages treated with the NF-κB inhibitor JSH-23 (25 μM) or GM1-dMOF@PTL (n = 3). Scale bar, 20 μm. (D-F) Corresponding quantitative analysis of ARG1, NOS2, and NLRP3 fluorescence intensity (n = 3). (G-I) Secretion levels of IL1B, IL18, and TNF in the supernatants of P65-OE macrophages (n = 5). (J) Schematic illustration of the in vitro Transwell co-culture system designed to evaluate the pathogenic paracrine crosstalk between macrophages (upper chamber) and resident structural cells (lower chamber). (K-N) Relative mRNA expression levels of the anabolic marker *Col2a1* and the catabolic marker *Mmp13* in (K, L) NPCs and (M, N) chondrocytes following co-culture. The recipient structural cells were either pre-treated with the specific TNFR antagonist R-7050 (5 μM) or co-cultured with M1 macrophages pre-treated with GM1-dMOF@PTL (n = 3). Data are presented as mean ± SD. The comparisons between groups were conducted using one-way ANOVA followed by Tukey’s post hoc test. *, *P* < 0.05; **, *P* < 0.01; ***, *P* < 0.001; ****, *P* < 0.0001.

**Figure S14**. GM1-dMOF@PTL reverses the catabolic phenotype in NPCs and chondrocytes in a co-culture system. (A) Relative mRNA expression of anabolic (*Acan, Col2a1*) and catabolic (*Mmp13, Adamts5*) markers in NPCs in the co-culture system (n = 3). (B) Relative mRNA expression of anabolic (*Acan, Col2a1*) and catabolic (*Mmp13, Adamts5*) markers in chondrocytes in the co-culture system (n = 3). Data are presented as mean ± SD. Statistical comparisons were performed using one-way ANOVA followed by Tukey’s post hoc test. **P*<0.05, ***P*<0.01, ****P*<0.001, *****P*<0.0001.

**Figure S15**. GM1-dMOF@PTL alleviates locomotor deficits and nociceptive hypersensitivity in a mouse model of OA. (A–E) OFT analysis quantifying locomotor deficits, including active time, distance travelled, central mean speed, and mean speed in each treatment group (n = 5). (F) Assessment of thermal hyperalgesia via measurement of PWL over 8 weeks (n = 5). (G) Assessment of mechanical allodynia via measurement of PWT over 8 weeks (n = 5). The Celecoxib-treated group served as a positive control for analgesia. Data are presented as mean ± SD. The comparisons between groups were conducted using one-way ANOVA followed by Tukey’s post hoc test. Statistical analysis of the longitudinal behavioral data was conducted using a two-way ANOVA followed by Tukey's post hoc test. *, *P* < 0.05 (OA + GM1-dMOF@PTL vs OA + dMOF@PTL); **, *P* < 0.01 (OA + GM1-dMOF@PTL vs OA + dMOF@PTL); ##, *P* < 0.01 (OA+GM1-dMOF@PTL vs OA+PTL); ###, *P* < 0.001 (OA+GM1-dMOF@PTL vs OA+PTL).

Supplementary table 1. The primers for mice used in the study.

| Genes | Forward (5’–3’) | Reverse (5’–3’) |
| --- | --- | --- |
| *Asc* | ACTTCTGTGACCCTGGCAATGAG | GCTGAGCAGCTGCAAACGAC |
| *Il1b* | GTATGGGCTGGACTGTTTC | GCTGTCTGCTCATTCACG |
| *Il6* | GATCTACTCGGCAAACCTAGTG | AGTGTCCCAACATTCATATTGTC |
| *Ifng* | CTGATGGGAGGAGATGTCTACACT | AGGGACAGCCTGTTACTACCTGA |
| *Il18* | GCCATGTCAGAAGACTCTTGCGTC | GTACAGTGAAGTCGGCCAAAGTTGTC |
| *Nlrp3* | GAGTTCTTCGCTGCTATGT | ACCTTCACGTCTCGGTTC |
| *Tnf* | TGCCTCCTCTTTTGCTTATGTT | AGGTTCAGTGATGTAGCGACAG |
| *Nos2* | ACAGGAGGGGTTAAAGCTGC | TTGTCTCCAAGGGACCAGG |
| *Il10* | GGTTGCCAAGCCTTATCGGA | TTCAGCTTCTCACCCAGGGA |
| *Mrc1* | TCTTTGCCTTTCCCAGTCTCC | TGACACCCAGCGGAATTTC |
| *Arg1* | AGACAGCAGAGGAGGTGAAGAGTAC | GGTAGTCAGTCCCTGGCTTATGGT |
| *Acan* | GCTGGCTGACCAGACAGTCA | CCGGATTC CGTAGGTTCTCA |
| *Col2a1* | CAGGCCTCGCGGTGAG | GTTCTCCATCTCTGCCACG |
| *Mmp13* | TGATGGCACTGCTGACATCAT | TGTAGCCTTTGGAACTGCTT |
| *Adamts5* | CTTCAATCCTTACCAGCATCG | TTACCATGACCATCATCCAGG |
| *Gapdh* | GACACTGAGCAAGAGAGGCCCTA | TGGGATGGAAATTGTGAGGGA |

**Note:** *Asc, apoptosis-associated speck-like protein containing a CARD; Il1b, interleukin 1 beta; Il6, interleukin 6; Ifng, interferon gamma; Il18, interleukin 18; Nlrp3, NLR family pyrin domain containing 3; Tnf, tumor necrosis factor; Nos2, nitric oxide synthase 2; Il10, interleukin 10; Mrc1, mannose receptor C-type 1; Arg1, arginase 1; Acan, aggrecan; Col2a1, collagen type II alpha 1 chain; Mmp13, matrix metallopeptidase 13; Adamts5, ADAM metallopeptidase with thrombospondin type 1 motif 5; Gapdh, glyceraldehyde-3-phosphate dehydrogenase*

Supplementary table 2. Detailed clinical characteristics of the human subjects included in the study.

| **Patient ID** | **Groups** | **Diagnosis** | **Age (years)** | **Gender** | **Grade** | **Inflammatory Diseases** |
| --- | --- | --- | --- | --- | --- | --- |
| **Spinal Cohort (Nucleus Pulposus)** | | | | | | |
| NP-N1 | Control | TCS | 18 | M | I | Absent |
| NP-N2 | Control | Trauma | 20 | F | II | Absent |
| NP-N3 | Control | Trauma | 32 | M | II | Absent |
| NP-N4 | Control | TCS | 40 | F | I | Absent |
| NP-N5 | Control | Trauma | 51 | M | II | Absent |
| NP-D1 | IVDD | LDH | 33 | F | IV | Absent |
| NP-D2 | IVDD | LDH | 40 | M | V | Absent |
| NP-D3 | IVDD | LDH | 49 | F | IV | Absent |
| NP-D4 | IVDD | LDH | 57 | M | V | Absent |
| NP-D5 | IVDD | LDH | 65 | F | V | Absent |
| **Knee Cohort (Articular Cartilage)** | | | | | | |
| AC-N1 | Control | Trauma | 22 | M | 0 | Absent |
| AC-N2 | Control | Trauma | 28 | F | 0 | Absent |
| AC-N3 | Control | Trauma | 32 | M | 1 | Absent |
| AC-N4 | Control | Trauma | 36 | F | 0 | Absent |
| AC-N5 | Control | Trauma | 41 | M | 1 | Absent |
| AC-O1 | OA | OA | 51 | F | 3 | Absent |
| AC-O2 | OA | OA | 59 | F | 4 | Absent |
| AC-O3 | OA | OA | 64 | M | 4 | Absent |
| AC-O4 | OA | OA | 70 | F | 3 | Absent |
| AC-O5 | OA | OA | 74 | F | 4 | Absent |

**Note:** *Radiological grades were assessed preoperatively based on MRI for the spine (Pfirrmann grading system: Grades I-II represent healthy degenerated discs; Grades IV-V represent severely degenerated discs) and X-ray for the knee (Kellgren-Lawrence (KL) grading system: Grades 0-1 represent healthy narrowing; Grades 3-4 represent moderate to severe osteoarthritis). NP: Nucleus Pulposus; AC: Articular Cartilage; IVDD: Intervertebral Disc Degeneration; OA: Osteoarthritis; LDH: Lumbar Disc Herniation; TCS: Tethered Cord Syndrome. All patients were screened to confirm the absence of confounding systemic inflammatory diseases such as Rheumatoid Arthritis and Ankylosing Spondylitis.*

**Supplementary Information of western blot images**
